# Development of a Hypersensitive Periodate-Cleavable Amino Acid that is Methionine- and Disulfide-Compatible and its Application in MHC Exchange Reagents for T Cell Characterisation

**DOI:** 10.1002/cbic.201200540

**Published:** 2012-12-23

**Authors:** Alessia Amore, Kim Wals, Evelyn Koekoek, Rieuwert Hoppes, Mireille Toebes, Ton N M Schumacher, Boris Rodenko, Huib Ovaa

**Affiliations:** a Division of Cell Biology, The Netherlands Cancer InstitutePlesmanlaan 121, 1066 CX Amsterdam (the Netherlands) E-mail: b.rodenko@nki.nlh.ovaa@nki.nl; b Division of Immunology, The Netherlands Cancer InstitutePlesmanlaan 121, 1066 CX Amsterdam (the Netherlands)

**Keywords:** amino acids, antigen presentation, cleavable linkers, immunoassays, peptides

## Abstract

Incorporation of cleavable linkers into peptides and proteins is of particular value in the study of biological processes. Here we describe the synthesis of a cleavable linker that is hypersensitive to oxidative cleavage as the result of the periodate reactivity of a vicinal amino alcohol moiety. Two strategies directed towards the synthesis of a building block suitable for solid-phase peptide synthesis were developed: a chemoenzymatic route, involving l-threonine aldolase, and an enantioselective chemical route; these led to α,γ-diamino-β-hydroxybutanoic acids in diastereoisomerically mixed and enantiopure forms, respectively. Incorporation of the 1,2-amino alcohol linker into the backbone of a peptide generated a conditional peptide that was rapidly cleaved at very low concentrations of sodium periodate. This cleavable peptide ligand was applied in the generation of MHC exchange reagents for the detection of antigen-specific T cells in peripheral blood cells. The extremely low concentration of periodate required to trigger MHC peptide exchange allowed the co-oxidation of methionine and disulfide residues to be avoided. Conditional MHC reagents hypersensitive to periodate can now be applied without limitations when UV irradiation is undesired or less practical.

## Introduction

Cleavable linkers are key tools in many fields of biology, ranging from proteomics and protein purification to structural biology. The development of linkers that can be cleaved under biocompatible reaction conditions is a challenge for chemists. A high cleavage rate, efficiency and selectivity are desired, but protein structure and function must not be compromised. This demands mild cleavage conditions in an environment that is dictated by the application (physiological or biochemical, in vivo or in vitro, reducing or pH-neutral, for example). A cleavable linker that can replace an amino acid residue in the backbone of a (poly)peptide or protein will allow cleavage of the backbone into at least two fragments upon application of a defined trigger, but will require accessibility of the cleavage site to the trigger. To date, various cleavable moieties that can be cleaved by photoirradiation, enzymes, nucleophilic or electrophilic reagents and reducing or oxidizing reagents have been developed for incorporation into the backbones of peptides or proteins.^1^ Although photocleavable linkers are widely used, UV irradiation has several drawbacks, such as 1) the uncontrolled increase in temperature and concomitant evaporation of medium or buffer that can be caused by the heat generated by a UV lamp, 2) incomplete cleavage due to limited penetration of UV light into the sample medium, and 3) uneven UV exposure of wells in microtiter plates in high-throughput screening applications, leading to distorted assay results. A chemical trigger circumvents these problems, is readily available, can be added and quenched in a controlled fashion and under sterile conditions and does not require special laboratory equipment such as a UV lamp. Linkers that can be cleaved under mild oxidative conditions have an advantage over linkers sensitive to reductive conditions: they allow preservation of disulfide bonds and hence protein structure.

The vicinal diol moiety has been used as an oxidation-sensitive linker^1^ and is cleaved by the mild oxidant sodium periodate to yield two aldehyde fragments. Periodate-mediated diol cleavage is a mild and biocompatible reaction, but it has one major drawback: complete cleavage of a diol linkage requires periodate concentrations in the millimolar range and incubation times of the order of hours. This invariably leads to co-oxidation of cysteine and methionine residues.^2^ Such co-oxidations can affect protein function and protein–ligand and/or protein–protein interactions.^3^

**Scheme 1:**
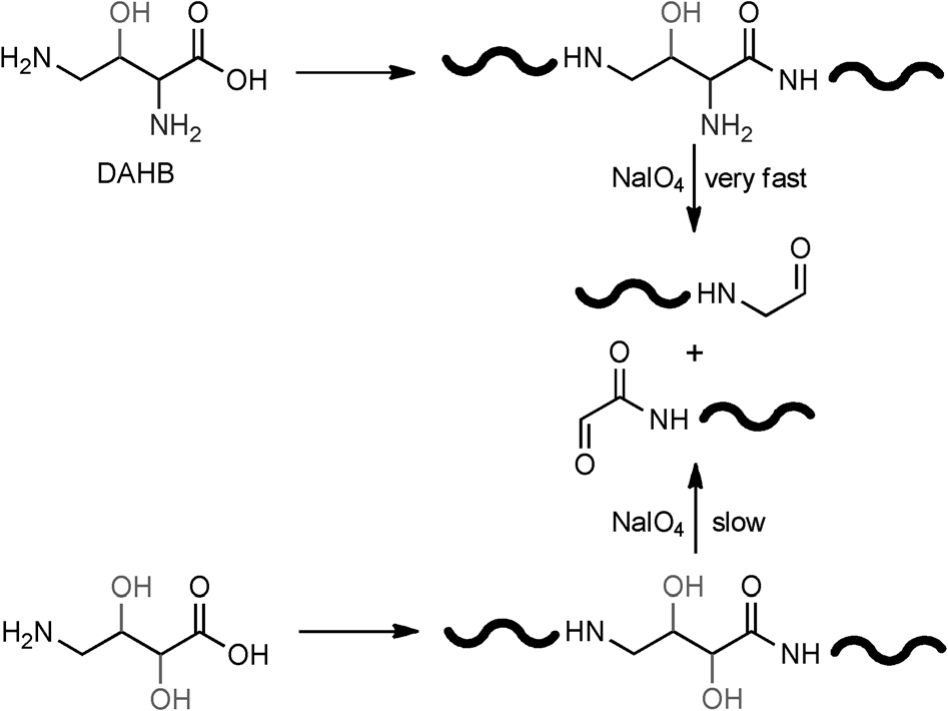
An α,γ-diamino-β-hydroxybutanoic acid (DAHB) residue incorporated into a peptide backbone is cleaved by NaIO_4_ much more rapidly than a γ-amino-α,β-dihydroxybutanoic acid residue.

We decided to develop a biocompatible, cleavable linker that would be amenable to incorporation into a (poly)peptide backbone and would also be hypersensitive to periodate oxidation, allowing selective cleavage, but without unwanted co-oxidation reactions. The periodate-mediated cleavage rate of a vicinal amino alcohol is reportedly 1000 times higher than that of a vicinal diol,^4^ so we aimed at the generation of a linker system containing a 1,2-amino alcohol fragment as shown in Scheme 1. Here we present the synthesis and stringent evaluation of this linker in a biological system in which the prevention of methionine oxidation is of critical importance: the major histocompatibility complex (MHC) class I–antigenic peptide–T-cell receptor interaction. Major histocompatibility class I complexes present peptide antigens at the cell surface for surveillance by cytotoxic T cells, forming the basis for a subsequent antigen-specific cytotoxic T cell response. T-cell receptors recognise the composite surface of the antigenic peptide-MHC class I complex (pMHC). Small changes in this surface, such as oxidation of a methionine residue in the antigenic peptide, result in distorted interaction with the T-cell receptor, and this has led to impaired T cell staining with use of MHC tetramers^5^ generated by MHC exchange reactions.^6^ We show that the vicinal amino alcohol linker developed here circumvents these co-oxidation issues in MHC exchange technology, which has become a standard tool for immunologists monitoring T cell specificities in a high-throughput fashion.

## Results

### Chemoenzymatic synthesis of 1,2-amino-alcohol-containing β-amino acids

**Scheme 2:**
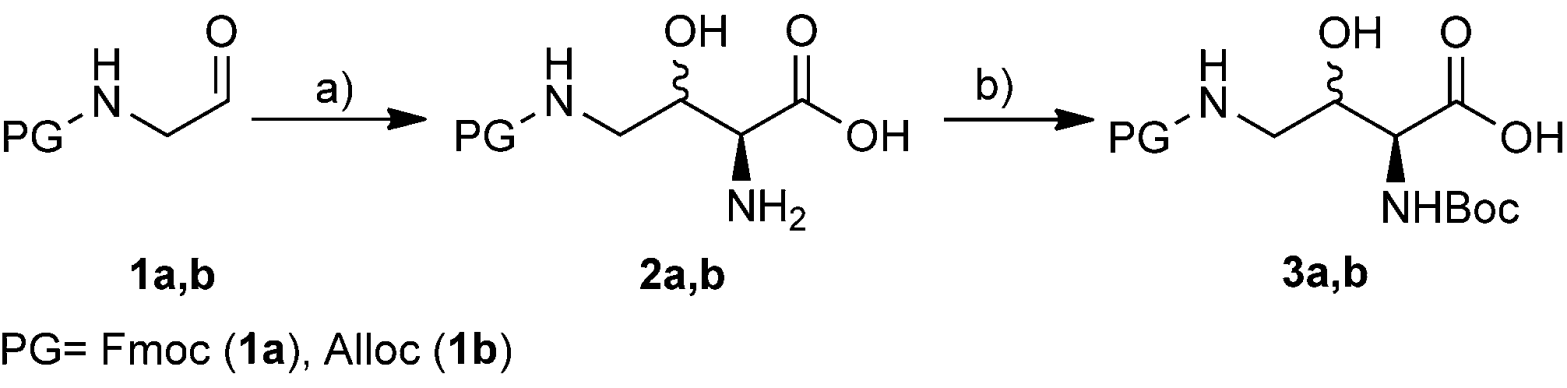
Chemoenzymatic synthesis of l-DAHB building blocks for SPPS. a) LTA lysate, glycine (2.7 equiv), pyridoxal-5′-phosphate (0.025 equiv), RT, 3–16 h, 10 % for **2 a** and 50 % for **2 b**; b) (Boc)_2_O (1.1 equiv), NaHCO_3_ (2 equiv), H_2_O/dioxane, 53 % for **3 a** and 40 % for **3 b**.

Although various synthetic routes for the preparation of vicinal amino alcohols—such as oxyamination^7^ or dihydroxylation^8^ of olefins, ring opening of aziridine^9^ and enzymatic processes^10^—have been developed, none of them allows incorporation of a vicinal amino alcohol group in the main carbon chain, so they are unsuited as synthetic approaches to the preparation of a backbone-cleavable amino acid residue. To generate the amino acid residue α,γ-diamino-β-hydroxybutanoic acid (DAHB), suitable for solid-phase peptide synthesis (SPPS), we used two different approaches: a biocatalytic route that gives the desired vicinal amino alcohol moiety in only few steps as a mixture of diastereoisomers [(2*S*,3*R*)- and (2*S*,3*S*)-DAHB] and, alternatively, a chemical route that requires more synthetic steps, but allows easy scale-up and furnishes only one diastereoisomer [(2*S*,3*R*)-DAHB]. Furthermore, to allow for easy incorporation in the backbone of a peptide ligand by automated solid-phase synthesis, we included orthogonal protective groups in the design of functionalised DAHB building blocks. The chemoenzymatic approach involved the application of an aldol reaction catalysed by recombinant l-threonine aldolase (LTA) from *Pseudomonas putida* (Scheme 2). This aldolase catalyses aldol condensations between glycine and appropriate aldehydes, with formation of C–C bonds with high stereoselectivity at the α-carbons to give the l-epimers. Although LTA from *Pseudomonas putida* is known to render modest enantioselectivity at the β-carbon chiral centres, we selected this enzyme for its tolerance of a broad range of aldehydes.10a, 11 LTA from *P. putida* was cloned and expressed in *E. coli*, and lysate containing LTA was used without further purification (Figure S1 in the Supporting Information). We studied the efficiency of the biocatalysed reaction with 2-aminoacetaldehyde bearing Fmoc (compound **1** **a**) or Alloc (compound **1** **b**) as N-protecting groups; these compounds were easily prepared by literature procedures.^12^ Enzymatic aldol condensations were performed at room temperature in aqueous phosphate buffer, with 2.7 equivalents of glycine and catalytic amounts of pyridoxal-5′-phosphate as the cofactor. The reactions were monitored by TLC and LC-MS and quenched by addition of CH_3_OH/AcOH (9:1, *v*/*v*) to avoid retroaldol reactions. Solubilisation of starting material **1** **a** required addition of DMSO (12 %) to the reaction mixture. After purification over a solid-phase extraction column, amino alcohol **2** **a** was obtained in 10 % yield. The presence of the organic solvent seemed to affect the efficiency of the enzymatic reaction: when *N*-Alloc-protected **1** **b** was used as the aldehyde component, DMSO was not required for its solubilisation and the reaction proceeded smoothly in phosphate buffer to give **2** **b** in good yield (50 %) after flash chromatography.

Next, the Nα atoms in **2** **a** and **2** **b** were protected with acid-labile Boc groups for subsequent use in Fmoc-based SPPS; l-DAHB derivatives **3** **a** and **3** **b** were obtained in 53 and 40 % yields, respectively. Maintaining the pH between 8 and 9 during the Boc protection step was essential to avoid retroaldol reactions, which are favoured at pH>9. The peaks of the *syn* and *anti* isomers of **3** **a** were discernible by HPLC and the *syn*/*anti* ratio was 40:60 (Figure S2), a typical diastereomeric ratio for aldol reactions catalysed by *P. putida* LTA.^13^ For products **2** **a**, **2** **b** and **3** **b** the *syn* and *anti* isomers were not resolved by HPLC, and their diastereomeric ratios could not be determined. Protection of the β-hydroxy groups was not necessary, because O-acylation does not occur with use of PyBop as the coupling reagent in standard Fmoc-based SPPS.

### Enantioselective synthesis of a β-amino acid containing a 1,2-amino alcohol system

**Scheme 3:**
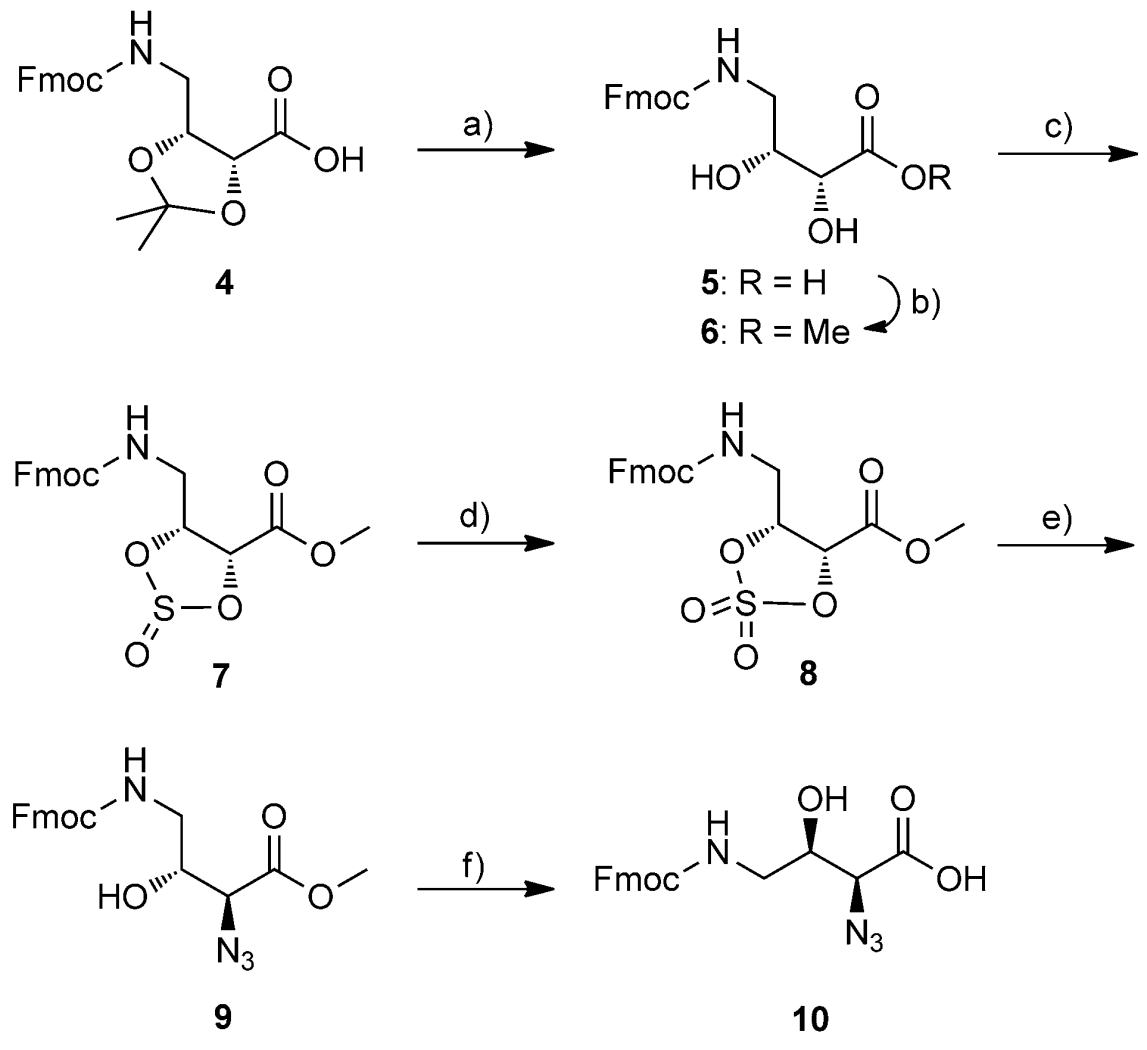
Enantioselective synthesis of a masked DAHB building block for SPPS. Reagents and conditions: a) TFA, THF/H_2_O, 4 h; b) CH_3_OH, HCl (cat), 65 °C, 6 h, 77 % over two steps; c) SOCl_2_ (2.2 equiv), Et_3_N (4.4 equiv), CH_2_Cl_2_, 0 °C, 1.5 h, 80 %; d) RuCl_3_**⋅***x* H_2_O (0.01 equiv), NaIO_4_ (2 equiv), CCl_4_/CH_3_CN/H_2_O (1:1:1.2), RT, 1 h, 80 %; e) NaN_3_ (2 equiv), acetone/H_2_O, 0 °C→RT, 16 h, 64 %; f) Me_3_SnOH, DCE, 82 °C, 3 h, 68 %.

The diastereoisomers of building blocks **3** obtained by the chemoenzymatic route were difficult to separate. In order to study the requirement for diastereoselectivity with regard to the periodate susceptibility of an amino alcohol moiety conformationally restrained in a peptide, we developed an alternative, enantioselective route to building blocks containing vicinal amino alcohol systems; this additionally gave access to different and orthogonal protective groups and allowed for easy scale-up (Scheme 3). Isopropylidene-protected vicinal diol **4**3b was treated with TFA in a THF/H_2_O mixture to furnish diol **5**, which, after solvent evaporation, was immediately taken up in methanol containing a catalytic amount of HCl to furnish the corresponding methyl ester **6** in 77 % yield. Treatment of diol **6** with thionyl chloride and triethylamine at 0 °C then gave sulfite **7** in 80 % yield. Direct ring opening of the sulfite was unsuccessful, so compound **7** was oxidized with NaIO_4_ and a catalytic amount of RuCl_3_⋅*x* H_2_O to afford sulfate **8** in 80 % yield. Nucleophilic ring opening of **8** with sodium azide was performed in an acetone/water mixture at room temperature to provide azido alcohol **9** in 64 % yield after column chromatography. Finally, mild hydrolysis with trimethyltin hydroxide^14^ furnished the desired masked amino alcohol building block **10**—in which the α-amino group was masked as an azide, to be reduced after incorporation into the peptide—in 68 % yield and as the single l-*syn* isomer.

With building blocks *syn*/*anti*-**3** **a**/**3** **b** and *syn*-**10** in hand we set out to synthesise a chemocleavable peptide containing an amino alcohol system as a conditional ligand for MHC exchange applications. Reagent accessibility is a key determinant in the efficiency of periodate cleavage and subsequent antigen exchange.3b The crystal structures of human leukocyte antigen-A2.1 (HLA-A2.1) loaded with various viral epitopes,^15^ including native cytomegalovirus (CMV) pp65_(495–503)_ peptide,^16^ or NLVJMVATV in which J is a diol residue,3b show that the p4 position is the most solvent-accessible position. We therefore incorporated the masked amino acid residues **3** **a**, **3** **b** and **10** at the p4 position in the HLA-A2.1 restricted CMV pp65_(495–503)_ epitope, resulting in NLVBMVATV, in which B designates the amino alcohol residue. By automated Fmoc-based SPPS we generated conditional peptide p*AA both as a *syn*/*anti* mixture of diastereomers (termed p*AA^*syn*/*anti*^) by utilizing *syn*/*anti*-**3** **a**/**3** **b** and as a single diastereoisomer (termed p*AA^*syn*^) from *syn*-**10**.

### Chemosensitivity and MHC exchange reactions

The sensitivity of p*AA towards sodium periodate was studied and compared with that of the diol-containing peptide analogue (termed p*DI). The oxidation-sensitive peptides were subjected to various amounts of NaIO_4_ and the resulting mixtures were analysed by LC-MS (Figures S3 and S4). Complete cleavage of the hypersensitive p*AA was achieved within 10 min with use of only two equivalents of periodate; as expected, this was much faster and milder than the conditions required for cleavage of p*DI, which required at least 30 equivalents of NaIO_4_ and 2 h incubation time. Peptide p*AA contains a methionine residue, and we observed that partial methionine oxidation only occurred when p*AA was treated with >3 equiv of NaIO_4_; this indicates that methionine oxidation was slower than amino alcohol cleavage.

**Figure 1:**
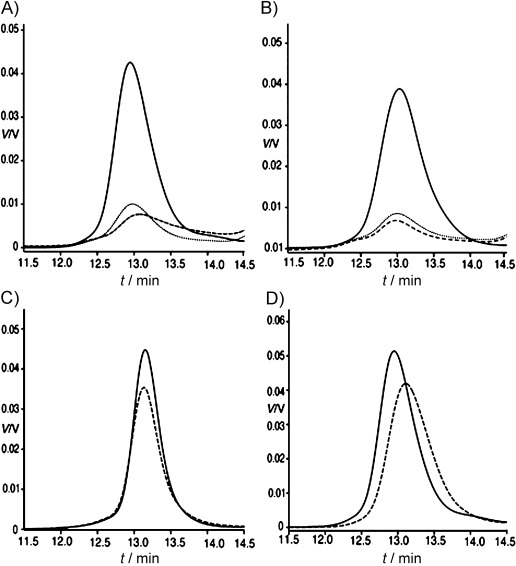
Peptide exchange efficiency as monitored by gel filtration HPLC analysis and UV absorbance (230 nm). A) HLA-A2.1::p*AA^*syn*/*anti*^ (0.5 μm) before (solid line) or after treatment with either 10 μm (dashed line) or 30 μm (dotted line) NaIO_4_ in the presence of 50 μm of nonbinding peptide (HLA-B7 restricted mage-1_(289–298)_ epitope RVRFFFPSL). B) As in A), but with 0.5 μm HLA-A2.1::p*AA^*syn*^. C) Chemo-exchange of 0.5 μm HLA-A2.1::p*AA^*syn*/*anti*^ before (solid line) or after treatment with 10 μm NaIO_4_ (dashed line) in the presence of 50 μm of rescue peptide (HLA-A2 restricted CMV pp65_(495–503)_ epitope NLVPMVATV). D) As in C), but with 0.5 μm HLA-A2.1::p*AA^*syn*^.

We next loaded p*AA^*syn*^ or p*AA^*syn*/*anti*^ into the MHC class I peptide binding groove of HLA-A2.1 by a standard refolding procedure.^20^ Chemocleavage of the conditional peptide lodged in the peptide binding groove should demand accessibility of the amino alcohol moiety to periodate anions, as well as an orientation of the vicinal amino alcohol that sterically allows the formation of the cyclic periodate ester intermediate.^17^ In view of the constrained conformation of the peptide ligand in the MHC groove, we investigated (by HPLC analysis) whether the loaded ligands p*AA^*syn*^ or p*AA^*syn*/*anti*^ would show differential reactivities towards periodate. Treatment of HLA-A2.1::p*AA^*syn*/*anti*^ or HLA-A2.1::p*AA^*syn*^ with as little as 10 μm NaIO_4_ in the presence of a nonbinding HLA-B7 restricted mage-1 epitope resulted in degradation of the conditional MHC complex (p*MHC) as a result of efficient oxidative cleavage (Figure 1 A and B). A peptide exchange experiment with HLA-A2 restricted CMV epitope, triggered by 10 μm NaIO_4_, resulted in rescue of the MHC complex (Figure 1 C and D). No difference in reactivity between MHC loaded with p*AA^*syn*^ or with p*AA^*syn*/*anti*^ was observed, which implied that the conformational restrictions in the MHC peptide binding groove have no appreciable influence on cleavage of the conditional peptide.

### MHC exchange reactions and T cell characterisation

We used refolded and biotinylated HLA-A2.1::p*AA^*syn*/*anti*^ for MHC exchange reactions and converted these MHC reagents into tetramers by addition of phycoerythrin-conjugated (PE-conjugated) streptavidin. We studied the performance of these hyper-chemosensitive MHC exchange reagents in the detection of antigen-specific T cells in peripheral blood samples by flow cytometry.

All exchange reactions mentioned from this point onwards were additionally performed with refolded and biotinylated HLA-A2.1::p*AA^*syn*^; the T cell staining results were similar to those obtained with biotinylated HLA-A2.1::p*AA^*syn*/*anti*^ (Supporting Information). This confirms that diastereomeric purity of the amino alcohol component is not a requirement for efficient MHC peptide exchange reactions.

**Figure 2:**
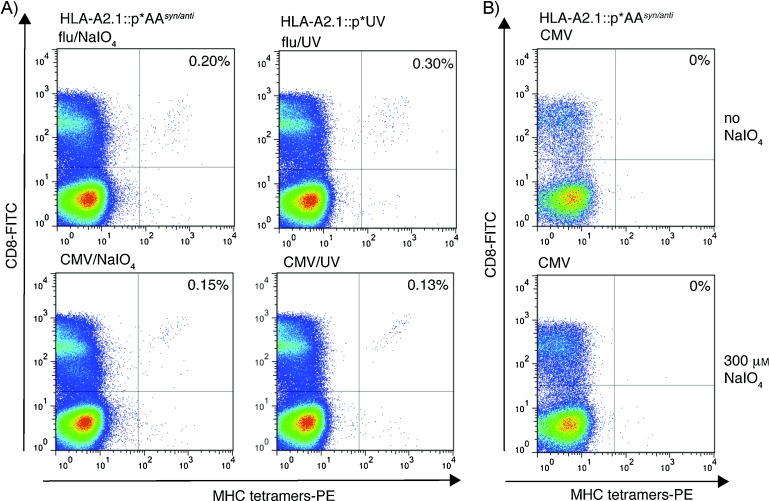
Staining of PBMCs of healthy donors with HLA-A2.1 exchange tetramers obtained by use either of 10 μm NaIO_4_ or of UV-mediated exchange as indicated. A) MHC peptide exchange with flu (influenza A matrix1_(58–66)_ epitope GILGFVFTL, top panels) or CMV (CMV pp65_(495–503)_ epitope NLVPMVATV, bottom panels). p*UV: KILGFVFJV, in which J is a photocleavable 3-amino-3-(2-nitrophenyl)propionic acid residue.^22^ B) MHC exchange with CMV before (top panel) and after (bottom panel) treatment with a large excess of NaIO_4_. Numbers indicate percentages of MHC tetramer^+^ cells amongst CD8^+^ cells.

Peptide exchange reactions with HLA-A2.1::p*AA were triggered by the addition of only 10 μm NaIO_4_, a concentration 30 times lower than that required with diol-based HLA-A2.1::p*DI. We observed that periodate-exchanged MHC tetramers containing influenza epitope GILGFVFTL stain low-frequency influenza epitope-specific T cells in peripheral blood mononuclear cells (PBMCs) from a healthy individual as efficiently as photo-exchanged MHC class I tetramers (Figure 2 A and Figure S8). Importantly, hypersensitive periodate-exchanged tetramers containing CMV epitope NLVPMVATV stain low-magnitude PBMCs from an HLA-A2.1-positive individual very efficiently, which implies that methionine oxidation, which would hamper interaction with the T-cell receptor, does not occur under these mild conditions. In contrast, treatment with 300 μm of NaIO_4_—the conditions needed for chemo-exchange of the diol-based p*MHC—impeded T cell staining, due to concomitant oxidation of the methionine residue (Figure 2 B). Staining of PBMCs with MHC tetramers obtained by hypersensitive chemo-exchange was not donor-dependent (Figure S9).

**Figure 3:**
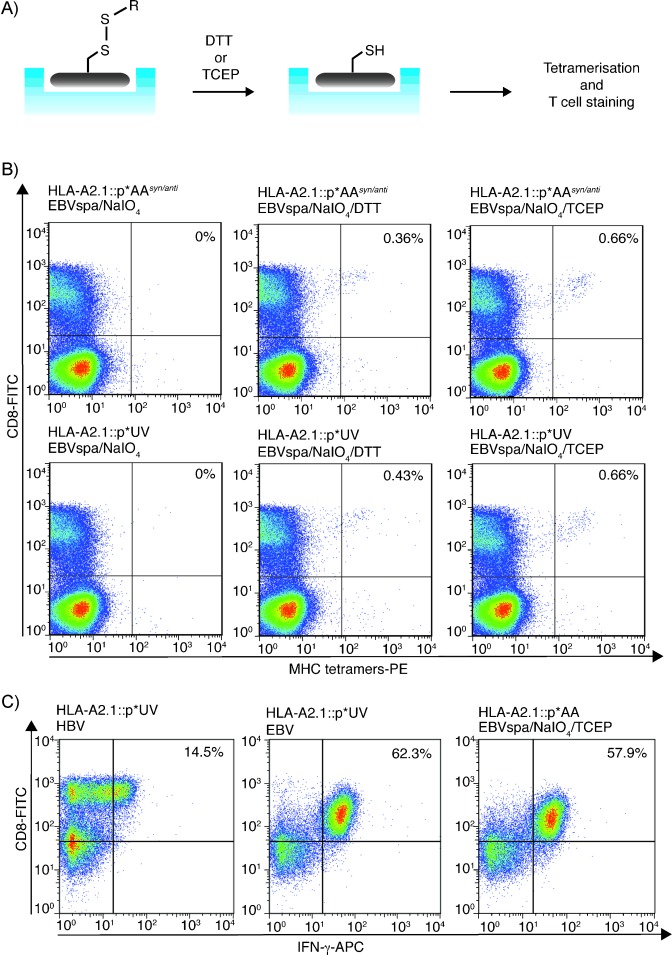
A) Concept of cysteine caging and liberation of the native antigenic epitope in the MHC peptide binding groove under mild conditions. R=SPa. B) Staining of PBMCs of healthy donors with HLA-A2.1 exchange tetramers obtained by use either of 10 μm NaIO_4_ or of UV-mediated exchange as indicated. EBV-SPa: EBV BMLF I_(259–267)_ epitope GLC(SPa)TLVAML, in which cysteine is caged with a thiopropanoic acid group. Numbers indicate the percentages of MHC tetramer^+^ cells of CD8^+^ cells before (left) and after addition either (middle) of 0.5 mm DTT or (right) of 0.5 mm TCEP. C) Stimulation of clonal CD8^+^ T cells specific to the EBV BMLF I_(259–267)_ epitope in complexation with HLA-A2.1 by direct interaction with the indicated exchanged MHC complexes immobilised on a streptavidin-coated plate. Similar levels of interferon-γ (IFN-γ) production, indicating T cell activation, were observed with the use of EBV-loaded tetramers obtained by UV exchange (middle panel) or with the use of chemo-exchange with EBV-Spa followed by TCEP-mediated Cys uncaging. HBV: HLA-A2.1 restricted hepatitis B virus core_(18–27)_ epitope, used here as a control for basal IFN-γ levels of nonactivated T cells (left panel).

MHC tetramers generated by the hypersensitive chemoexchange reaction of HLA-A2.1::p*AA with Epstein–Barr virus (EBV) epitope GLCTLVAML, containing both an oxidation-sensitive Cys residue and an oxidation-sensitive Met residue, did not stain PBMCs from an HLA-A2.1/EBV-positive individual (Figure S10). We attributed the impaired performance of these chemo-exchange tetramers to the presence of the cysteine residue. Isosteric replacement, a work-around that we have used before, gave variable, donor-dependent results in T cell staining by chemo-exchanged tetramers.3b We therefore turned to another strategy: cysteine caging, which has been made feasible by the extremely mild oxidative cleavage conditions used here, which preclude disulfide oxidation.^18^ This concept is based on protecting the cysteine residue in the epitope in the form of a disulfide that is liberated, after hypersensitive chemo-exchange, by a mild reducing reagent such as dithiothreitol (DTT) or tris(2-carboxyethyl)phosphine (TCEP), thereby regenerating a free cysteine residue without compromising MHC integrity (Figure 3 A). The cysteine residue of the EBV epitope was caged as a thiopropanoic acid disulfide, termed EBV-SPa (Figure S5). Exchange tetramers containing EBV-SPa did not stain peripheral blood cells of an EBV-positive donor, but treatment of EBV-Spa-exchanged monomers with either DTT or TCEP followed by tetramerisation resulted in efficient detection of EBV-positive cytotoxic T lymphocytes (Figure 3 B and Figure S11).

We also investigated the use of thiomethyl and thio-*tert*-butyl disulfides as caging groups, but these gave suboptimal results (Figures S6, S7, S12 and S13). To show the functionality of T cells interacting with chemo-exchanged multimers we performed a cytokine (IFN-γ) release assay, which revealed that chemo-exchanged multimers containing an uncaged EBV-Spa epitope activate an EBV-positive T-cell clone as efficiently as UV-exchanged multimers (Figure 3 C).

## Discussion

We have developed synthetic routes—one giving a diastereomeric mixture and one enantiopure—to vicinal amino alcohol building blocks for application in SPPS. If diastereomeric purity is not essential, as was the case for periodate-triggered MHC peptide exchange, we recommend the use of the racemic *syn*/*anti*-l-DAHB building blocks **3** **a** (*N*-Fmoc-protected) or **3** **b** (*N*-Alloc-protected), given their ease of synthesis (only two steps from the protected 2-aminoacetaldehydes). The higher yields obtained in the synthesis of *N*-Alloc-protected **3** **b** would favour the use of this building block. However, if such yields are of no concern, the direct applicability of *N*-Fmoc-protected **3** **a** in standard Fmoc-based SPPS protocols is a clear advantage.

Since its introduction in 2006,6a MHC exchange technology has become a standard tool for immunologists studying T cell selectivity and reactivity, and over 65 publications describing the use of exchange tetramers have appeared to date. Although the majority of conditional MHC ligands reported to date are based on UV cleavage of a 3-amino-3-(2-nitrophenyl)propionic acid residue, one can envisage that for certain applications UV irradiation would be less practical. Uniform parallel irradiation, as required for high-throughput screening of large collections of potential antigens, for example, is often difficult to achieve, which in turn yields skewed assay results. In addition, evaporation accelerated by the heat generated by the UV lamp is problematic when working with minute volumes in, for example, pMHC microarrays,^19^ or in other situations in which reagents are a limiting factor. In early attempts we tried to address these issues by the development of an orthogonal chemocleavable MHC exchange ligand, based on the reactivity of vicinal diols towards sodium periodate.3b Sodium periodate concentrations of at least 300 μm were required to effectuate peptide exchange, however, and these conditions appeared incompatible with exchange peptides containing oxidation-prone residues such as Met or Cys. Recovery of the impaired T cell staining by utilization of nonoxidizable Met and Cys carba-isosteres was only partially successful, because the tolerance of these bioisosteres by the relevant T-cell receptor appeared to be donor-dependent, thus limiting the general applicability of this methodology.

The vicinal amino alcohol building block that we present here conveniently solved these problems because the peptide—p*AA—incorporating this moiety and loaded into the MHC complex was cleaved at a very low NaIO_4_ concentration (10 μm), which precludes Met co-oxidation. T cell staining difficulties with Cys-containing exchange peptides were simply overcome by temporarily caging the Cys residue as a disulfide during the very mild oxidative exchange reaction. Subsequent uncaging by a mild reductant (DTT or TCEP) released the native MHC-bound epitope, eventually resulting in T-cell-reactive exchange tetramers. Notably, Cys disulfide caging has been made possible by the extremely mild oxidative conditions that enabled cleavage of the 1,2-amino alcohol. A disulfide-caged Cys residue can easily be incorporated in the exchange peptide during SPPS by making use of the corresponding protected building block. Although N-terminal Ser and Thr residues remain prone to periodate cleavage,2b these residues can be replaced by other periodate-resistant ones, because the N-terminal residue of an MHC class I epitope is not critical to interactions with the T-cell receptor.

## Conclusions

In conclusion, the development of a vicinal amino alcohol building block has made periodate-mediated peptide backbone cleavage possible under extremely mild conditions and without accompanying methionine and disulfide oxidation. As a representative application we have shown that this cleavable moiety has truly opened chemically triggered MHC exchange technology to the broader scientific community, taking away the need for special laboratory equipment and allowing MHC class I epitope screening and assessment of T cell reactivities without limitations. Furthermore, we can also envisage the application of the vicinal amino alcohol building block in other conditional settings, such as when triggered depletion of effector peptides or ring-opening of cyclic peptides is desired. Moreover, incorporation of this building block into a protein by native chemical ligation, leading to a protein that can be cleaved at will by application of a mild chemical stimulus, is an interesting possibility.

## Experimental Section

**General**: All commercial materials (Aldrich, Fluka, Novabiochem) were used without further purification. Peptide synthesis reagents (standard amino acid building blocks and PyBop) were purchased from Novabiochem. All solvents were reagent grade or HPLC grade. Unless stated otherwise, reactions were performed under inert atmospheres. NMR spectra (^1^H and ^13^C) were recorded with a Bruker Avance 300 spectrometer, referenced to TMS or residual solvent. LC-MS analysis was performed with a system containing a Waters 2795 separation module (Alliance HT), Waters 2996 Photodiode Array Detector (190–750 nm), Waters Alltima C18 (2.1×100 mm) or Phenomenex Kinetex XB-C18 (2.1×50 mm) reversed-phase column and a Micromass LCT-TOF mass spectrometer. Samples were run at 0.40 mL min^−1^ (Waters C18) or 0.80 mL min^−1^ (Kinetex C18) with use of a gradient of two mobile phases: A) aq. formic acid (0.1 %), and B) formic acid in CH_3_CN (0.1 %). Data processing was performed with the aid of Waters MassLynx 4.1 software. Preparative HPLC was performed with a Shimadzu LC-20AD/T instrument fitted with a C18 Vydac column (Grace Davison Discovery Sciences) with use of gradient elution [mobile phases: A) aq. TFA (0.05 %) and B) TFA in CH_3_CN (0.05 %)]. Gel-filtration chromatography was performed with a Biosep SEC S3000 column (Phenomenex) in PBS (pH 7.4).

**Cloning and expression of l-threonine aldolase**: l-Threonine aldolase DNA from *P. putida* (accession number AB001577.1) was codon-optimised for expression in *E. coli* and commercially obtained from GeneArt. The 1041-base-pair DNA fragment was then amplified by PCR with use of primers 5′-GGTGGT CATATG ATGACC GATCAG AGCC and 5′-GGTGGT GGATCC CTAGTG ATGGTG ATGGTG ATGCGC GCTCAT CACC. The PCR product was inserted between the NdeI and BamHI restriction sites of the commercially available pet3a vector, which yielded plasmid pRHH1 (5699 bp). *E. coli* BL21 (DE3) pLysS cells were transformed with pRHH1 and expression was carried out at 37 °C in Luria–Bertani (LB) medium supplemented with chloramphenicol (34 μg mL^−1^) and carbenicillin (50 μg mL^−1^). Cells were grown to an OD_600nm_ value of 0.6 and protein expression was induced by addition of isopropyl β-D-thiogalactopyranoside to a final concentration of 1 mm. After 4 h, the cells were harvested by centrifugation at 4 °C at 4000 *g* for 15 min and taken up in PBS buffer. Cells were then sonicated and the lysate was spun at 4 °C at 4000 *g* for 15 min to remove cell debris. The supernatant containing LTA (39 kDa) was directly used for the synthetic steps described below.

**(2*S*)-2-Amino-4-({[(9*H*-fluoren-9-yl)methoxy]carbonyl}amino)-3-hydroxybutanoic acid (2 a)**: A solution of **1 a**^12^ (280 mg, 1 mmol) in DMSO (2 mL) and a solution of glycine (200 mg, 2.7 mmol) in KH_2_PO_4_ buffer (pH 8.0, 50 mm, 2.6 mL) were added at 0 °C to KH_2_PO_4_ buffer (pH 8.0, 50 mm, 10 mL). LTA lysate (1 mL of 10 mg protein mL^−1^) and pyridoxal-5′-phosphate (5 mm in H_2_O, 75 μL) were added, and the mixture was placed on a horizontal shaker. After the mixture had been shaken at room temperature for 16 h, the reaction was quenched by addition of methanol/AcOH (95:5), lowering the pH to 3. Et_2_O was then added to the mixture, and after the mixture had been stirred for 5 min, the ether layer was removed. The remaining mixture was filtered over a Strata SCX cation-exchange solid-phase extraction column (Phenomenex) with elution with methanol/acetonitrile (1:1) and lyophilized to afford **2 a** in 10 % yield. N.B. The product gradually decomposes in organic solvents and should be stored dry at −20 °C or immediately used for the next step. ^1^H NMR (300 MHz, DMSO): *δ*=7.90 (d, *J*=7.9 Hz, 2 H), 7.72–7.68 (m, 2 H), 7.45–7.31 (m, 4 H), 4.36–3.90 (m, 5 H), 3.20–3.17 ppm (m, 2 H); ^13^C NMR (300 MHz, DMSO): *δ*=168.64, 156.35, 143.35, 140.65, 127.59, 127.05, 125.15, 120.07 68.24, 65.56, 46.64, 30.65 ppm; HRMS (ESI): calcd: 357.3725 [*M*+H]^+^; found: 357.1323.

**(2S)-4-{[(Allyloxy)carbonyl]amino}-2-amino-3-hydroxybutanoic acid (2 b)**: Glycine (300 mg, 4 mmol) was added to a solution of **1 b** (150 mg, 1 mmol) in KH_2_PO_4_ buffer (pH 8.0, 50 mm, 1 mL), followed by the addition of LTA (1 mL) and pyridoxal-5′-phosphate (5 mm in H_2_O, 75 μL). The resulting mixture was placed on a horizontal shaker for 3 h. The reaction was quenched by addition of methanol/AcOH (95:5), lowering the pH to 3. The mixture was lyophilised and then purified by flash chromatography (eluents: *n*-butanol/H_2_O/AcOH 2:1:0.5) to afford **2 b** in 50 % yield. ^1^H NMR (300 MHz, D_2_O): *δ*=5.97–5.84 (m, 1 H), 5.30–5.81 (m, 2 H), 4.53–4.52 (m, 2 H), 4.12–4.06 (m, 1 H), 3.76 (d, *J*=3.5 Hz, 0.45 H), 3.60 (d, *J*=4.3 Hz, 0.48 H), 3.33–3.18 ppm (m, 2 H); ^13^C NMR (300 MHz, D_2_O): *δ*=172.99, 171.88, 132.66, 117.45, 68.76, 66.06, 57.27, 56.89 (minor diastereoisomer), 43.34, 42.68 ppm (minor diastereoisomer).

**(2*S*)-2-[(*tert*-Butoxycarbonyl)amino]-4-({[(9*H*-fluoren-9-yl)methoxy]carbonyl}amino)-3-hydroxybutanoic acid (3 a)**: Compound **2 a** (67 mg, 0.19 mmol) was dissolved in water/dioxane/methanol (20:1:1, *v*/*v*/*v*) and cooled to 0 °C, and solid NaHCO_3_ (62 mg, 0.76 mmol) was added to the suspension. A solution of di-*tert*-butyl dicarbonate (45 mg, 0.21 mmol) in dioxane was added dropwise, and the mixture was stirred at 4 °C for one hour. (N.B. The pH should be kept between 8 and 9, to avoid the retroaldol reaction, which takes place at pH>9.) The reaction was quenched by lowering the pH to 3 by addition of KHSO_4_ (1 m). The solution was partly concentrated by cold evaporation, and the water phase was extracted twice with cold ethyl acetate. The combined organic layers were washed with brine, dried with Na_2_SO_4_ and concentrated to dryness in vacuo to afford the crude product, which was purified by flash chromatography (CH_2_Cl_2_/methanol 9:1) to afford **3 a** as a white glassy solid (47 mg, 53 %). ^1^H NMR (300 MHz, CD_3_OD): *δ*=7.69 (d, *J*=7.7 Hz, 2 H), 7.55 (d, *J*=7.5 Hz, 2 H), 7.29–7.18 (m, 4 H), 4.25–4.11 (m, 5 H), 3.20–3.19 (m, 2 H), 1.37 and 1.35 ppm (2 s, 9 H, minor and major diastereoisomer, respectively); ^13^C NMR (300 MHz, CD_3_OD): *δ*=158.95, 158.44, 145.36, 146.60, 128.77, 128.16, 126.10, 120.91, 80.99, 80.96 (minor diastereoisomer), 72.11 (minor diastereoisomer), 71.29, 67.99, 58.06 (minor diastereoisomer), 57.09, 44.83, 28.68 ppm; MS (ESI): calcd: 478.17 [*M*+Na]^+^; found: 478.94.

**(2S)-4-{[(Allyloxy)carbonyl]amino}-2-[(*tert*-butoxycarbonyl)amino]-3-hydroxybutanoic acid (3 b)**: The procedure described for **3 a** was also used to prepare **3 b** in 40 % yield. ^1^H NMR (300 MHz, MeOD): *δ*=6.01–5.88 (m, 1 H), 5.35–5.18 (m, 2 H), 4.53–4.52 (m, 2 H), 4.26–3.95 (m, 2 H), 3.33–3.18 (m, 2 H), 1.47 ppm (s, 9 H); ^13^C NMR (300 MHz, MeOD): *δ*=174.27, 173.73, 158.80, 158.38, 134.42, 117.56, 80.96, 80.82 (minor), 66.57 (minor), 66.53, 61.93, 58.09 (minor), 57.34, 44.80, 44.64 (minor), 28.69 ppm.

**(2*R*,3*R*)-4-({[(9*H*-Fluoren-9-yl)methoxy]carbonyl}amino)-2,3-dihydroxybutanoic acid (5)**: TFA (1.3 mL) was added to a solution of **4**3b (1 g, 2.5 mmol) in THF/H_2_O (2.5:1 mL) and the mixture was stirred for 4 h at room temperature. The solvent was evaporated in vacuo to afford **5** (870 mg) as a white powder that was used as such for the next step. ^1^H NMR (300 MHz, DMSO): *δ*=7. 89 (d, *J*=7.6 Hz, 2 H), 7.77 (d, *J*=7.6 Hz, 2 H), 7.44–7.30 (m, 4 H), 7.15 (br, 1 H), 4.28–4.18 (m, 3 H), 3.91 (d, *J*=3.9 Hz, 1 H) 3.76–3.70 (m, 1 H), 3.25–3.01 ppm (m, 2 H); ^13^C NMR (300 MHz, DMSO): *δ*=173.73, 157.29, 143.89, 140.66, 127.57, 127.04, 125.24, 120.05, 72.90, 71.32, 65.44, 46.67, 43.11 ppm.

**Methyl (2*R*,3*R*)-4-({[(9*H*-fluoren-9-yl)methoxy]carbonyl}amino)-2,3-dihydroxybutanoate (6)**: Concentrated HCl (1 mL) was added to a solution of **5** (870 mg, 2.4 mmol) in methanol (100 mL) and the mixture was stirred at 65 °C for 6 h. The solvent was evaporated, and the crude product was purified by flash chromatography (ethyl acetate/hexanes 3:2→2:1) to afford **6** as a white solid (690 mg, 77 %). ^1^H NMR (300 MHz, CDCl_3_): *δ*=7.77 (d, *J*=7.6 Hz, 2 H), 7.59 (d, *J*=7.6 Hz, 2 H), 7.44–7.30, 5.20 (br, 1 H), 4.47 (d, *J*=6.5 Hz, 2 H), 4.24–4.23 (dd, *J*=6.5 Hz, 1 H), 4.14 (d, *J*=5.86 Hz, 1 H), 3.99–3.93 (m, 1 H) 3.84 (s, 3 H), 3.53–3.38 ppm (m, 2 H); ^13^C NMR (300 MHz, CDCl_3_): *δ*=173.31, 157.67, 143.79, 141.36, 127.76, 127.09, 124.95, 120.03, 72.16, 71.76, 67.01, 52.85, 47.23, 42.67 ppm.

**Methyl (4*R*,5*R*)-5-[({[(9*H*-fluoren-9-yl)methoxy]carbonyl}amino)methyl]-2-oxy-1,3,2-dioxathiolane-4-carboxylate (7)**: Et_3_N (1.1 mL, 8.14 mmol) and a solution of SOCl_2_ (0.3 mL, 4.1 mmol) in dry CH_2_Cl_2_ (2 mL) were added at 0 °C to a solution of **6** (690 mg, 1.85 mmol) in dry CH_2_Cl_2_ (11 mL). The resulting mixture was stirred at 0 °C until disappearance of the starting material was observed by TLC (about 1.5 h). The mixture was then poured into cold water and extracted with ethyl acetate. The organic layer was washed with water and brine and dried over Na_2_SO_4_. Concentration to dryness in vacuo yielded the crude product, which was purified by flash chromatography (ethyl acetate/Hex 1:1) to afford **7** as a yellow oil (630 mg, 80 %). ^1^H NMR (300 MHz, CDCl_3_): *δ*=7.79 (d, *J*=7.6 Hz, 2 H), 7.62 (d, *J*=7.6 Hz, 2 H), 7.58–7.33 (m, 4 H), 5.32–5.20 (m, 3 H), 4.49 (br s, 2 H), 3.78 (s, 3 H), 3.74–3.65 (m, 1 H), 3.44–3.34 ppm (m, 1 H).

**Methyl (4*R*,5*R*)-5-[({[(9*H*-fluoren-9-yl)methoxy]carbonyl}amino)methyl]-1,3,2-dioxathiolane-4-carboxylate 2,2-dioxide (8)**: H_2_O (6 mL), RuCl_3_**⋅***x* H_2_O (4.5 mg, 0.02 mmol) and NaIO_4_ (643 mg, 3 mmol) were added at 0 °C to a solution of **7** (630 mg, 1.5 mmol) in CCl_4_/CH_3_CN 10 mL (1:1, *v*/*v*). After the slurry had been stirred for 1 h at 0 °C, Et_2_O (30 mL) and hexanes (30 mL) were added. The organic layer was separated, filtered over filter paper and washed with aq. NaHCO_3_ (1 %). The water phase was back-extracted with Et_2_O, and the combined organic layers were washed with brine, dried with Na_2_SO_4_ and concentrated to dryness in vacuo to afford **8** as a white powder (470 mg, 70 %). ^1^H NMR (300 MHz, CDCl_3_): *δ*=7.70 (d, *J*=7.6 Hz, 2 H), 7.50 (d, *J*=7.6 Hz, 2 H), 7.37–7.23 (m, 4 H), 5.25–5.06 (m, 2 H), 4.42 (br s, 2 H), 4.17–4.12 (dd, 1 H), 3.78 (s, 3 H), 3.62–3.49 ppm (m, 2 H); ^13^C NMR (300 MHz, CD_3_OD): *δ*=164.12, 156.15, 143.59, 141.46, 127.89, 127.21, 124.96, 120.06, 80.74, 77.54, 67.34, 53.73, 47.08, 40.34 ppm; FTIR (solid): 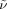
=1716, 1396, 1211 cm^−1^.

**Methyl (2*S*,3*R*)-2-azido-4-({[(9*H*-fluoren-9-yl)methoxy]carbonyl}amino)-3-hydroxybutanoate (9)**: NaN_3_ (130 mg, 2 mmol) was added at 0 °C to a solution of **8** (420 mg, 1 mmol) in acetone (4 mL) and water (0.5 mL), and the mixture was stirred at room temperature for 2 h. Cold H_2_SO_4_ (20 %) and Et_2_O (1:1, *v*/*v*, 10 mL) were added and stirring was continued for 16 h. Et_2_O was added, and the phases were separated. The water phase was washed with CH_2_Cl_2_. The organic phases were washed with water and brine. The organic layers were combined and evaporated in vacuo to afford **9** (280 mg, 64 %). ^1^H NMR (300 MHz, CDCl_3_): *δ*=7.75 (d, *J*=7.5 Hz, 2 H), 7.57 (d, *J*=7.4 Hz, 2 H), 7.42–7.29 (m, 4 H), 4.47 (d, *J*=6.5 Hz, 2 H), 4.23–4.19 (m, 2 H), 3.87–3.84 (m, 1 H), 3.37–3.28 ppm (m, 2 H); ^13^C NMR (300 MHz, CD_3_OD): *δ*=169.22, 157.79, 143.55, 141.34, 127.81, 127.16, 124.95, 120.01, 71.04, 67.65, 63.74, 53.27, 47.07, 44.24 ppm; FTIR (solid): 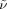
=2112, 1750, 1701, 1527, 1267 cm^−1^.

**(2*S*,3*R*)-2-Amino-4-({[(9*H*-fluoren-9-yl)methoxy]carbonyl}amino)-3-hydroxybutanoic acid (10)**: Trimethyltin (500 mg, 2.5 mmol) was added to a solution of **9** (200 mg, 0.5 mmol) in dichloroethane (6 mL) and the solution was stirred at 82 °C for 3 h. The mixture was allowed to cool to room temperature, and cold ethyl acetate (5 mL) was added, followed by cold aq. HCl (5 %, 5 mL). The mixture was stirred for 10 min, extra ethyl acetate was added, and the phases were separated. The organic layer was washed with water and brine, dried with Na_2_SO_4_ and concentrated to dryness in vacuo. The crude product was purified over a short silica column (CH_2_Cl_2_/methanol 98:2→95:5) to afford the pure product **10** as an off-white solid (130 mg, 68 %). ^1^H NMR (300 MHz, CD_3_OD): *δ*=7.85 (d, *J*=7.8 Hz, 2 H), 7.73 (d, *J*=7.8 Hz, 2 H), 7.42–7.29 (m, 4 H), 4.40 (d, *J*=7.0 Hz, 2 H), 4.24–4.20 (m, 2 H), 3.90–3.82 (m, 1 H), 3.28–3.23 ppm (m, 2 H); ^13^C NMR (300 MHz, CD_3_OD): *δ*=173.32, 159.78, 146.04, 143.37, 129.55, 128.94, 126.92, 121.72, 72.88, 68.50, 65.55, 49.24, 45.71 ppm; FTIR (solid): 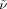
=3300, 2112, 1741, 1672, 1541 cm^−1^; MS (ESI): calcd: 382.1277 [*M*+H]^+^; found: 383.1122.

**Peptide synthesis**: All peptides were synthesised by standard Fmoc SPPS on a 25 μmol scale with a Syro II MultiSyntech automated peptide synthesiser. Removal of Alloc groups was achieved with Pd(PPh_3_)_4_ (3 equiv) in CH_2_Cl_2_/AcOH/*N*-methylmorpholine (37:2:1) mixtures. Azido groups were reduced by treatment with Bu_3_P (5 equiv) in THF containing a few droplets of water. Peptides were cleaved from the resin and deprotected with TFA/*i*Pr_3_SiH/H_2_O (95:2.5:2.5), precipitated in cold *n*-pentane/diethyl ether and purified by RP-HPLC (C18). All peptides were analysed by LC-MS and used at >98 % purity.

**Protein expression and purification**: MHC class I refolding reactions were performed as described, and class I complexes were purified by gel-filtration chromatography. Biotinylation and MHC tetramer formation were performed as described.^20^

**Protocol for hypersensitive MHC peptide exchange triggered by NaIO_4_**: Exchange peptide (pH 7.4, 50 μm) and biotinylated HLA-A2.1::p*AA (0.5 μm) in PBS (50 μL) were treated with NaIO_4_ (10 μm) for 45 min at room temperature. Optionally, subsequent uncaging of disulfide-protected Cys residues is achieved by treatment with DTT or TCEP (500 μm) for 15 min. Exchanged monomers were used for tetramerisation as previously described^20^ or for cytokine release assays as described below.

**T cells**: For analysis of MHC tetramer binding and T cell responses in human samples, peripheral blood mononuclear cells of healthy volunteers were obtained by Ficoll gradient separation. A cryopreserved CD8^+^ T-cell clone, derived from a healthy donor expressing the HLA-A2.1 complex specifically binding EBV BMLF I_(259–267)_, was kindly provided by Ms. Marit van Buuren (NKI, Amsterdam, NL). Cryopreserved cells were thawed 24 h prior to cytokine release assays and were suspended in cytotoxic T lymphocyte (CTL) medium [RPMI 1640 medium supplemented with Glutamax, HEPES, human serum (10 %) and penicillin/streptomycin (6000 U mL^−1^)].

**Tetramer staining**: Cells were stained with the indicated MHC tetramers for 4 min at 37 °C. Subsequently, cells were incubated with anti-CD8 antibody (BD Biosciences) for 10–15 min at 25 °C. Data acquisition and analysis was carried out with a FACSCalibur (Becton Dickinson) instrument and use of FlowJo software.

**Cytokine release assay**: T cell activity was analysed by an adapted procedure based on the use of immobilised MHC tetramers.^21^ Briefly, exchange monomers (100 μL per well) were added to a 96-well streptavidin coated plate (NUNC, Immobilizer Streptavidin, #436014) blocked with bovine serum albumin (BSA)/PBS (2 %). After 30 min incubation, the plate was washed with PBS. T cells were spun at 1700 rpm for 10 min and were resuspended in CTL medium supplemented with protein transport inhibitor (1 μL mL^−1^, GolgiPlug, BD Biosciences #555029) at 4×10^5^ cells mL^−1^. T cells were added (100 μL per well) to the MHC-streptavidin coated plate, and the plate was briefly centrifuged (700 rpm, 3 min) to enhance MHC/T cell contact before incubation for 4 h at 37 °C under CO_2_ (7 %). The cells were pelleted at 1700 rpm for 3 min, resuspended in FACS buffer [50 μL, BSA (0.5 %), NaN_3_ (0.02 %) in PBS] and transferred to a 96-well U-bottom plate (BD FALCON #353077). Cells were stained with FITC-conjugated antibodies to CD8 (BD Biosciences, #345772) for 15 min at 25 °C, fixed and permeabilised (BD Cytofix/Cytoperm kit, #554714), and stained with APC-conjugated antibodies to IFNγ (BD Biosciences, #341117) for 30 min at 4 °C. Samples were analysed by flow cytometry.

## Supplementary material

Detailed facts of importance to specialist readers are published as ”Supporting Information”. Such documents are peer-reviewed, but not copy-edited or typeset. They are made available as submitted by the authors.
