# Supplementary material for: Development of a Hypersensitive Periodate-Cleavable Amino Acid that is Methionine- and Disulfide-Compatible and its Application in MHC Exchange Reagents for T Cell Characterisation
Source: Chembiochem. 2012 Dec 23;14(1):123–31. doi: 10.1002/cbic.201200540 (PMC3561698; doi:10.1002/cbic.201200540)

CHEMBIOCHEM

Supporting Information

© Copyright Wiley-VCH Verlag GmbH & Co. KGaA, 69451 Weinheim, 2012

Development of a Hypersensitive Periodate-Cleavable Amino Acid that is Methionine- and Disulfide-Compatible and its Application in MHC Exchange Reagents for T Cell Characterisation

Alessia Amore,[a] Kim Wals,[a] Evelyn Koekoek,[a] Rieuwert Hoppes,[a] Mireille Toebes,[b] Ton N. M. Schumacher,[b] Boris Rodenko,*[a] and Huib Ovaa*[a]

cbic_201200540_sm_miscellaneous_information.pdf

Supporting information:

Contents	Page
**L-Threonine aldolase cloning and expression**	S2
**LC-MS analysis of building block 3a**	S3
**Periodate reactivity studies of (hyper)chemosensitive conditional peptides**	S4
**Cysteine disulfide caging**	S6
**Flow Cytometry**	S8
**1H and 13C NMR spectra of compounds 2a, 2b, 3a, 3b, 6, 7, 8, 9 and 10**	S12

L-Threonine aldolase cloning and expression

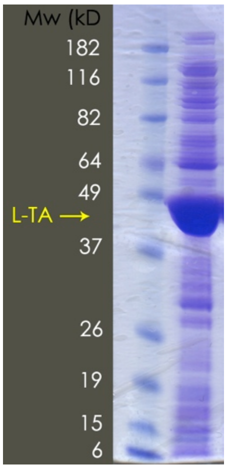

Figure S1. SDS-PAGE of a lysate of *E. coli* Bl21 (DE3) pLysS overexpressing L-threonine aldolase of *Pseudomonas putida*. The left lane contains a protein marker, the right lane contains the lysate that was used for subsequent aldol condensation reactions.

LC-MS analysis of building block 3a

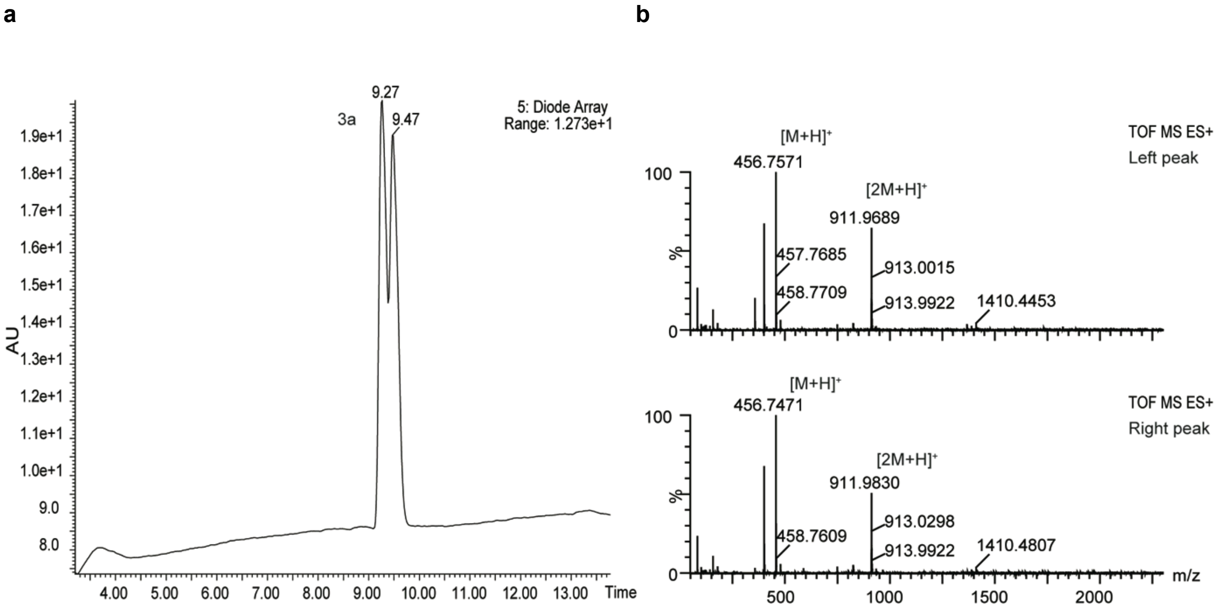

Figure S2. LC-MS analysis of building block L-**3a**, showing a mixture of L-*syn* and L-*anti* diastereomers. (a) C18-HPLC profile. (b) Mass spectrum of the indicated HPLC peaks.

Periodate reactivity studies of (hyper)chemosensitive conditional peptides

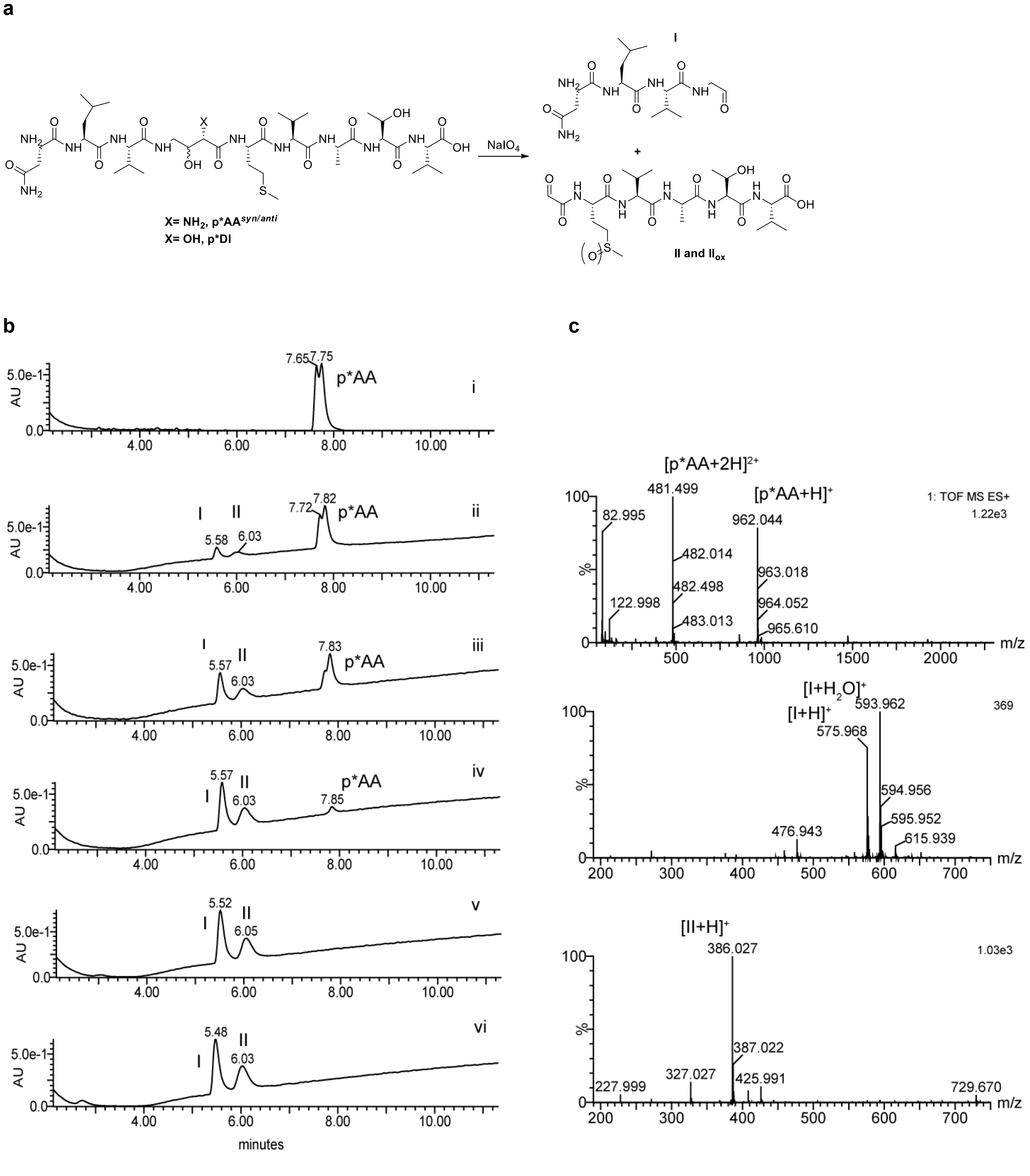

Figure S3. Chemosensitivity of peptide ligand **p*AA** towards $NaIO_4$. Peptide **p*AA** containing the vicinal amino alcohol linker was subjected to various amounts of $NaIO_4$ for 10 minutes. (a) Cleavage of **p*AA** leads to two fragments: a 3-mer aldehyde (I) and an N-substituted glyoxaldehyde 5-mer (II). (b) The cleavage mixtures were analyzed by LC-MS using NH_4OAc at neutral pH 7 as an elution buffer to avoid potential acid catalyzed methionine oxidation during the HPLC run. (b) LC-MS analysis of **p*AA** with $NaIO_4$ at room temperature for 10 minutes leading to fragments **I** and **II**. C18-HPLC profiles before (i) and after (ii-vi) treatment with $NaIO_4$: i) no $NaIO_4$; ii) 0.2 equiv; iii) 0.5 equiv; iv) 1 equiv; v) 2 equiv; vi) 3 equiv $NaIO_4$. (c) Mass spectra of the HPLC peaks designated **p*AA**, I and II.

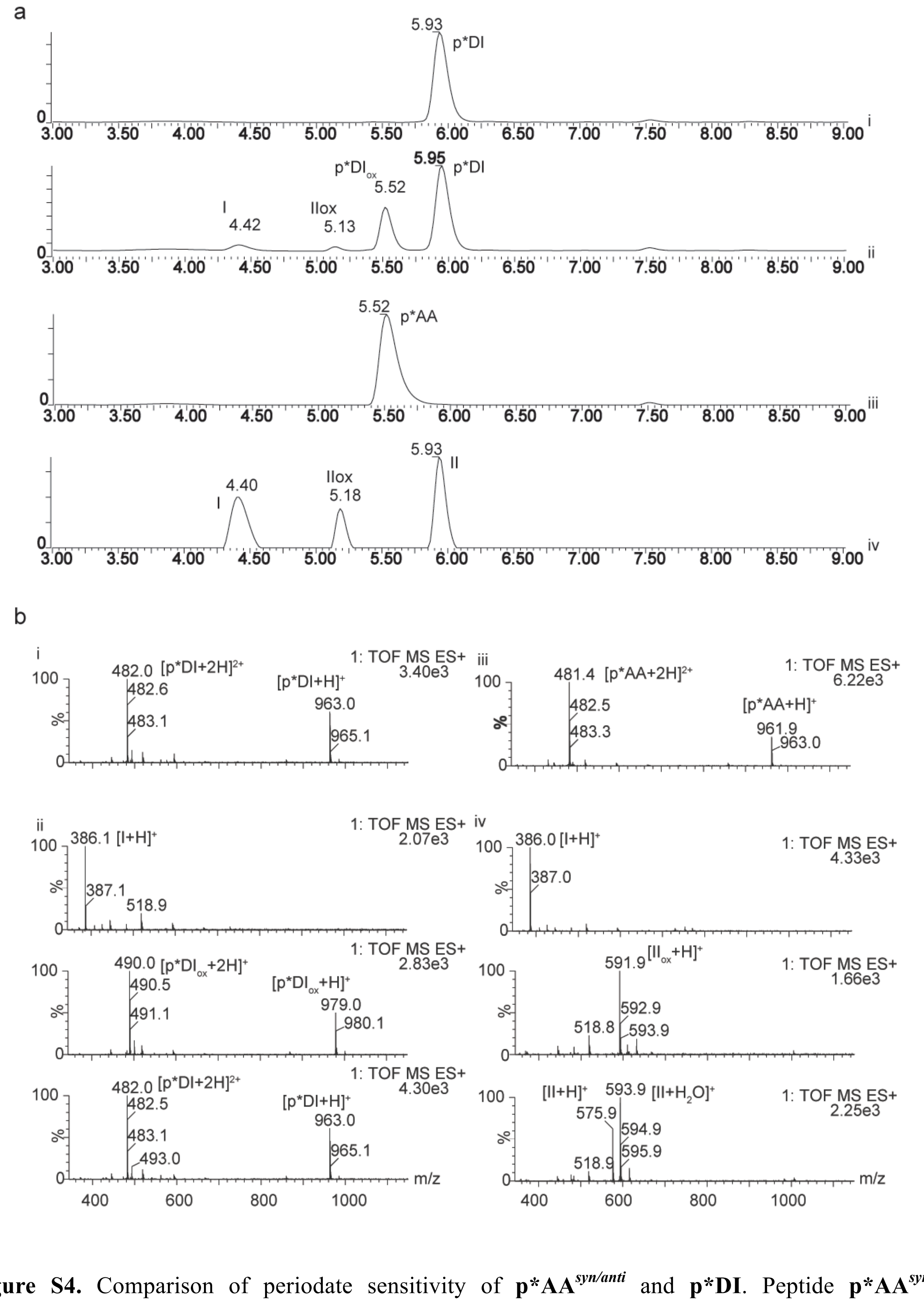

Figure S4. Comparison of periodate sensitivity of **p*AA**$^{syn/anti}$ and **p*DI**. Peptide **p*AA**$^{syn/anti}$ (containing the amino alcohol linker) and peptide **p*DI** (containing the diol linker) were subjected to $NaIO_4$ leading to a 3-mer aldehyde (**I**), an *N*-substituted glyoxaldehyde 5-mer (**II**) and, at higher $NaIO_4$ concentrations, methionine oxidized forms **p*DI$_{ox}$** and **II$_{ox}$**, see also Figure S3a. The peptides **p*AA**$^{syn/anti}$ or **p*DI** were treated with $NaIO_4$ (3 equiv) for 10 minutes and subsequently analyzed by LC-MS. (a) C18-HPLC profiles before and after treatment with $NaIO_4$, where i) **p*DI** control, no $NaIO_4$; ii) 3 equiv $NaIO_4$ for 10 min; iii) **p*AA**$^{syn/anti}$ control, no $NaIO_4$; iv) 3 equiv $NaIO_4$ for 10 min. (b) Mass spectra of the annotated HPLC peaks (left panels for profiles i and ii, right panels for profiles iii and iv).

Cysteine disulfide caging

Procedure for cysteine disulfide caging. DIPEA (1.1 equiv) was added to a solution of S-methyl methanesulfonothioate (3 equiv) in DMF, followed by the dropwise addition of peptide EBV BMLF $I_{(259\text{-}267)}$ GLCTLVAML (1 equiv). The resulting mixture was stirred under argon for 16 hours. Then, the solvent was removed and the crude product was purified by reversed phase HPLC to afford pure peptide EBV-SMe. MS (ESI): $[M+H]^+$ calcd, 966.49; found 966.25 (Figure S5).

EBV-SPa was generated via the protocol above using 3-((methylsulfonyl)thio)propanoic acid as the thiolating agent, MS (ESI): $[M+H]^+$ calcd, 1024.50; found 1024.21 (Figure S6).

EBV-tBu was obtained by Fmoc-based solid phase peptide synthesis using commercially available Fmoc-*S*-StBu-cysteine as a building block (Figure S7).

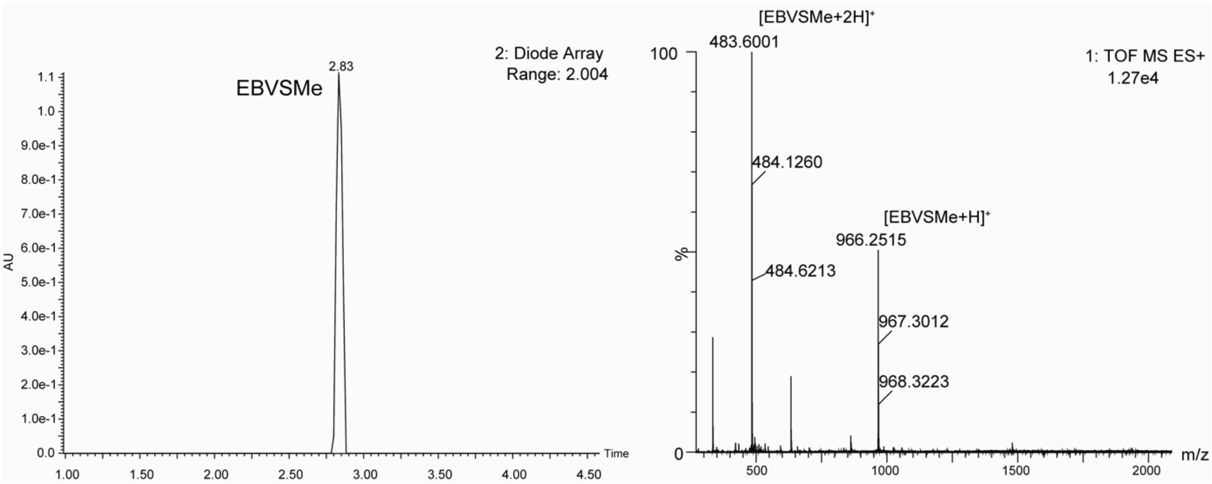

Figure S5. LC-MS analysis of EBV-SMe. C18-HPLC profile (left panel) and mass spectrum (right panel) of EBV-SMe GLC(SMe)TLVAML.

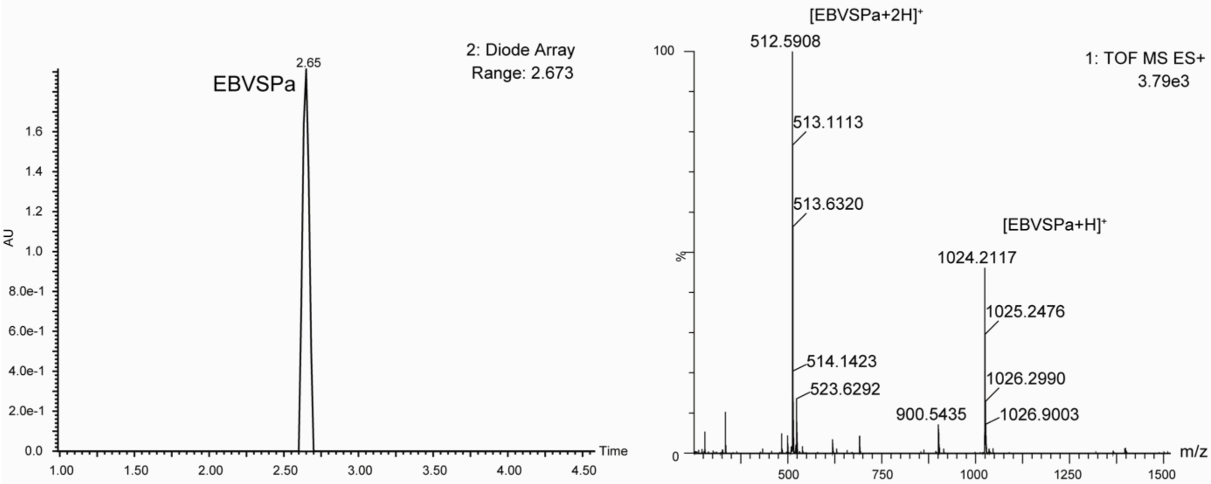

Figure S6. LC-MS analysis of EBV-SPa. C18-HPLC profile (left panel) and mass spectrum (right panel) of EBV-SPa GLC(SPa)TLVAML.

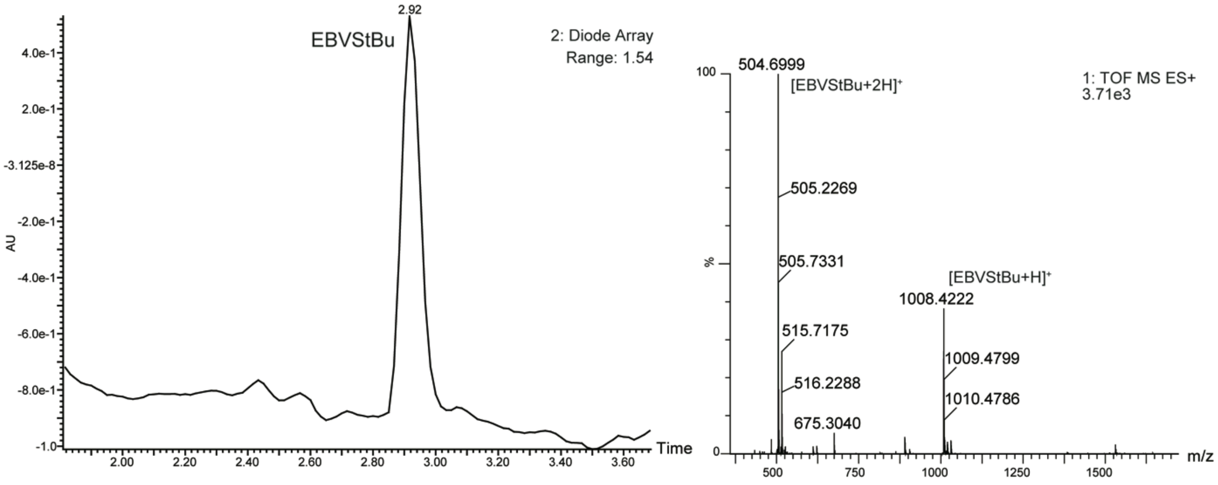

Figure S7. LC-MS analysis of EBV-StBu. C18-HPLC profile (left panel) and mass spectrum (right panel) of EBV-StBu GLC(StBu)TLVAML.

Flow Cytometry

For analysis of chemoexchanged MHC tetramer binding and T cell responses in human samples, peripheral blood mononuclear cells of healthy volunteers were obtained by Ficoll gradient separation. Cells were stained with the indicated MHC tetramers for 4 min at 37 °C. Subsequently, cells were incubated with anti-CD8 antibody (BD Biosciences) for 10-15 min at 25 °C. Data acquisition and analysis was carried out on a FACSCalibur (Becton Dickinson) instrument using FlowJo software. The conditional peptide referred to in all FACS plots as **p*AA** is NLVBMVATV, in which B designates the amino alcohol residue. The conditional peptide ligands are termed **p*AA**$^{syn/anti}$, when a *syn/anti* mixture of diastereoisomeric amino alcohol building block was incorporated, or **p*AA**^{syn}, when the single *syn*-diastereoisomer was incorporated; **p*UV** refers to KILGFVFJV, in which J is a photocleavable 3-amino-3-(2-nitrophenyl)propionic acid residue.

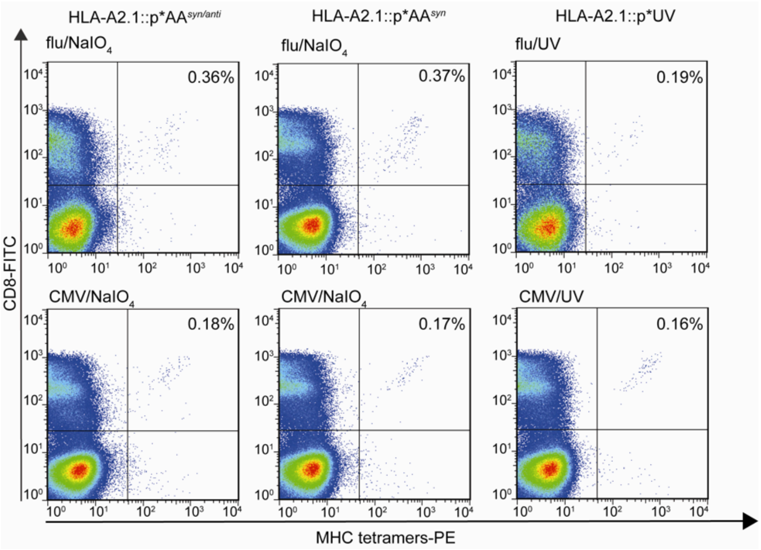

Figure S8. Hypersensitive chemoexchange tetramers efficiently stain low frequency antigen specific PMBCs. Staining of PBMC of healthy donors with HLA-A2.1 exchange tetramers obtained with either 10 µM $NaIO_4$ or UV mediated exchange as indicated. Flu: Influenza A Matrix-1$_{(58-66)}$ epitope GILGFVFTL; CMV: CMV pp65$_{(495-503)}$ epitope NLVPMVATV. Numbers indicate the percentage of MHC tetramer$^+$ cells amongst CD8$^+$ cells.

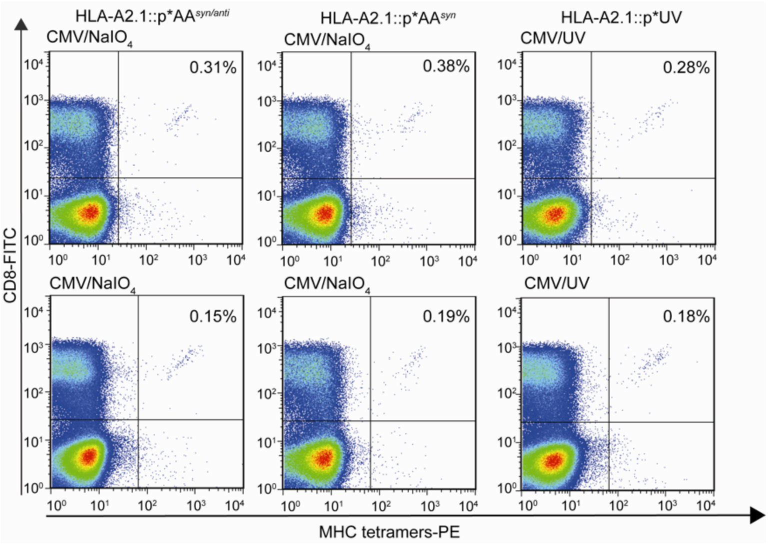

Figure S9. Staining of PBMCs by hypersensitive chemoexchange tetramers is not donor-dependent. Staining of PBMC of different healthy donors (donor A, top panels; donor B, bottom panels) with HLA-A2.1 exchange tetramers obtained with either 10 µM $NaIO_4$ or UV mediated exchange as indicated. CMV: CMV $pp65_{(495\text{-}503)}$ epitope NLVPMVATV. Numbers indicate the percentage of MHC tetramer$^+$ cells amongst CD8$^+$ cells.

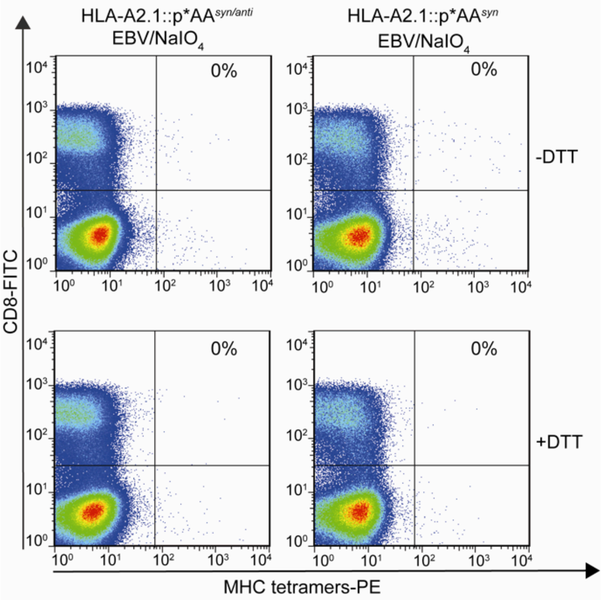

Figure S10. A free cysteine containing peptide is not compatible with hypersensitive chemoexchange and resulting tetramers lead to impaired T cell staining. Staining of PBMC of a healthy donor with HLA-A2.1 exchange tetramers obtained by 10 µM $NaIO_4$ exchange. EBV, EBV-BMLF $I_{(259\text{-}267)}$ GLCTLVAML epitope. Numbers indicate the percentage of MHC tetramer$^+$ cells of total CD8$^+$ cells before (top panels) and after (bottom panels) addition of 0.5 mM DTT.

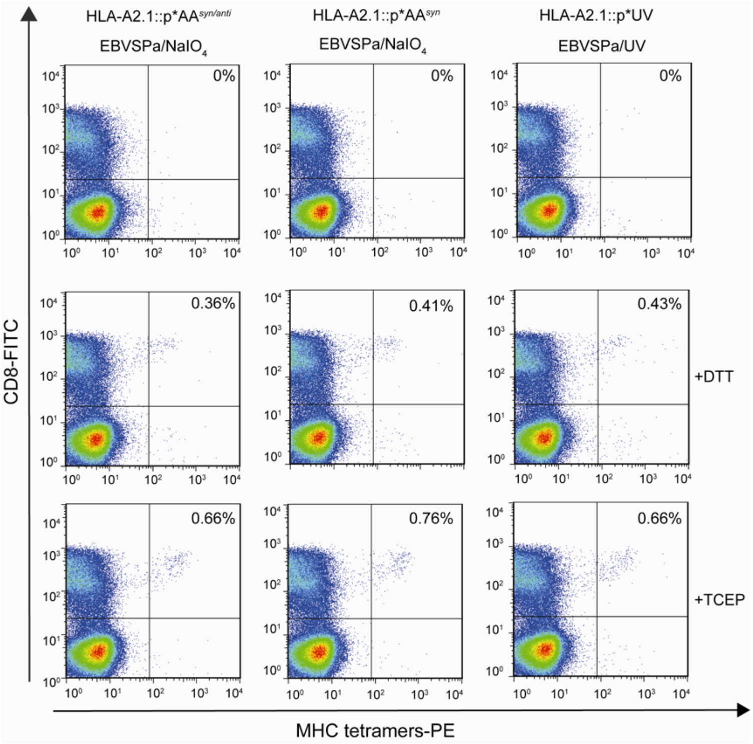

Figure S11. Hypersensitive chemoexchange tetramers loaded with a peptide containing a thiopropanoic acid disulfide caged cysteine residue result in efficient T cell staining following uncaging by mild reducing agents DTT or TCEP. Staining of PBMCs of a healthy donor with HLA-A2.1 exchange tetramers obtained by either 10 µM $NaIO_4$ or UV mediated exchange as indicated. EBVSPa, an EBV-BMLF $I_{(259\text{-}267)}$ epitope in which cysteine is caged as an SPa group. Numbers indicate the percentage of MHC tetramer$^+$ cells of total CD8$^+$ cells before (top panels) and after addition of 0.5 mM DTT (middle panels) or 0.5 mM TCEP (bottom panels).

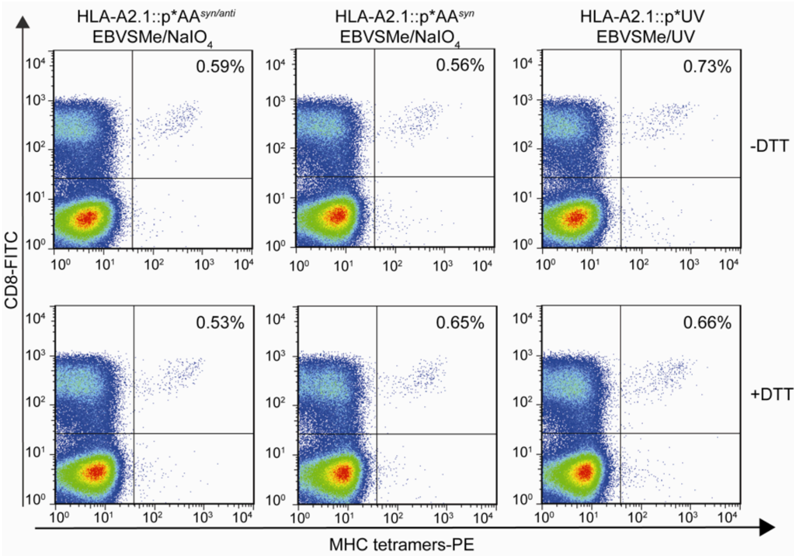

Figure S12. Hypersensitive chemoexchange tetramers loaded with a peptide containing a thiomethyl disulfide caged cysteine residue stain PBMCs also before DTT mediated uncaging, indicating that the thiomethyl moiety acts as cysteine bioisostere. Staining of PBMCs of a healthy donor with HLA-A2.1 exchange tetramers obtained by either 10 µM $NaIO_4$ or UV mediated exchange as indicated. EBV-SMe, EBV-BMLF $I_{(259\text{-}267)}$ epitope in which cysteine is caged as a thiomethyl group. Numbers indicate the percentage of MHC tetramer$^+$ cells of total CD8$^+$ cells before (top panels) and after (bottom panels) addition of 0.5 mM DTT.

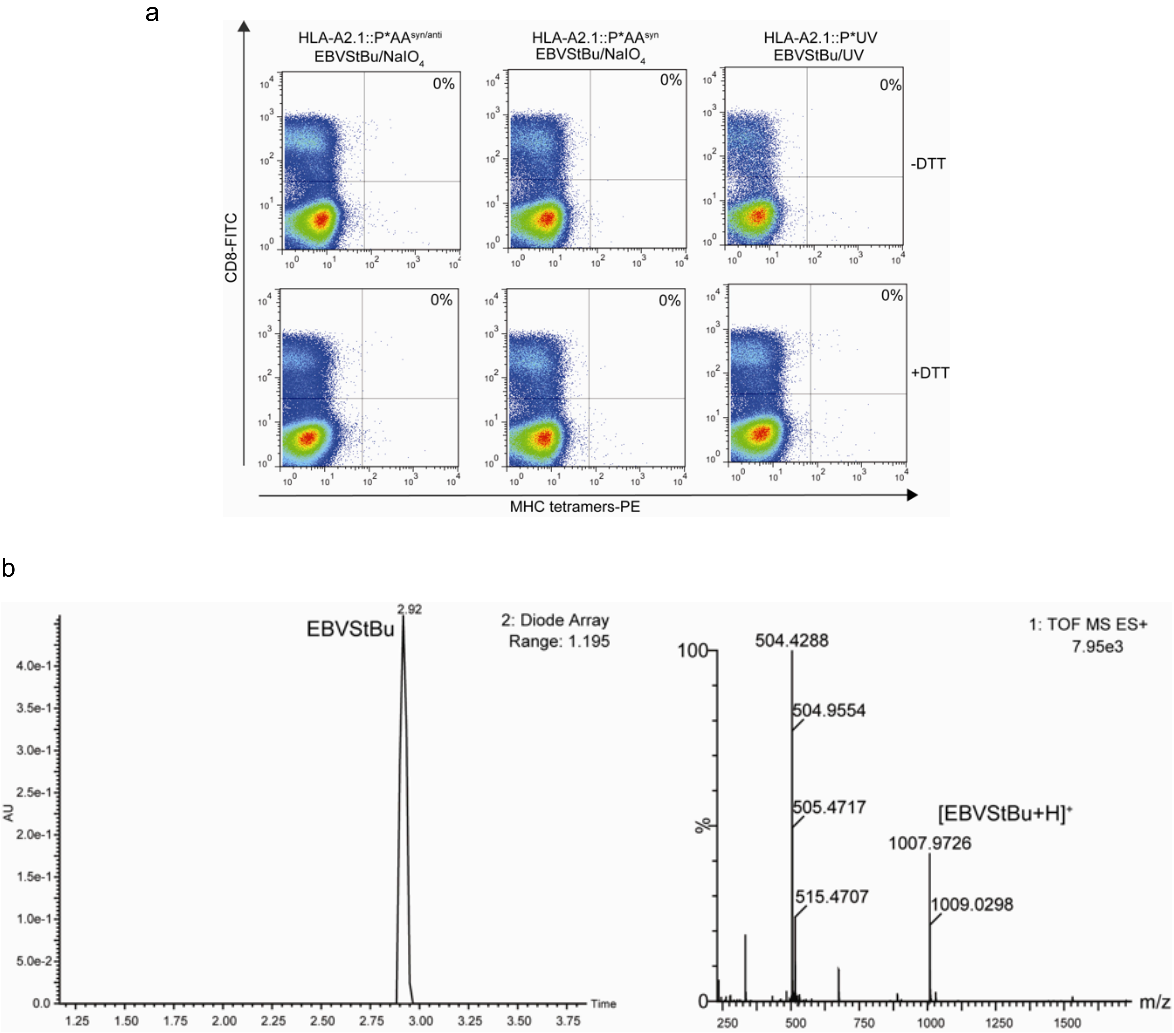

Figure S13. Hypersensitive chemoexchange tetramers loaded with a peptide containing a thio-*tert*-butyl disulfide caged cysteine residue do not stain PBMCs, nor following attempted DTT mediated uncaging, indicating that the thio-*tert*-butyl group is not removed under mild reducing conditions. (a) Staining of PBMCs of a healthy donor with HLA-A2.1 exchange tetramers obtained with either 10 μM $NaIO_4$ or UV mediated exchange as indicated. EBV-S*t*Bu, EBV-BMLF $I_{(259\text{-}267)}$ epitope in which cysteine is protected with a S*t*Bu group. Numbers indicate the percentage of MHC tetramer^{+} cells of total CD8^{+} cells before (top panels) and after (bottom panels) addition of DTT. (b) LC-MS analysis of EBV-tBu treated with DTT shows that uncaging of the EBV epitope does not occur. C18-HPLC profile (left panel) and mass spectrum (right panel) of 0.5 μM EBV-StBu treated with 0.5 mM DTT.

1H and 13C NMR spectra of compounds 2a, 2b, 3a, 3b, 6, 7, 8, 9 and 10.

Compound 2a

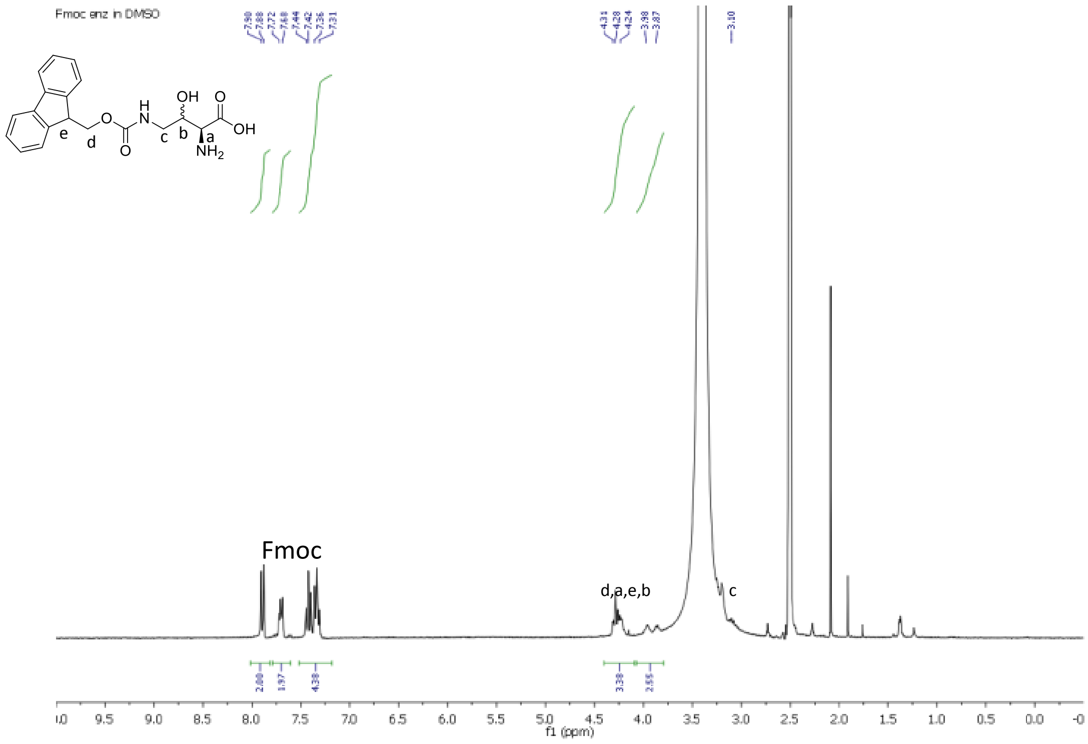

Compound 2b

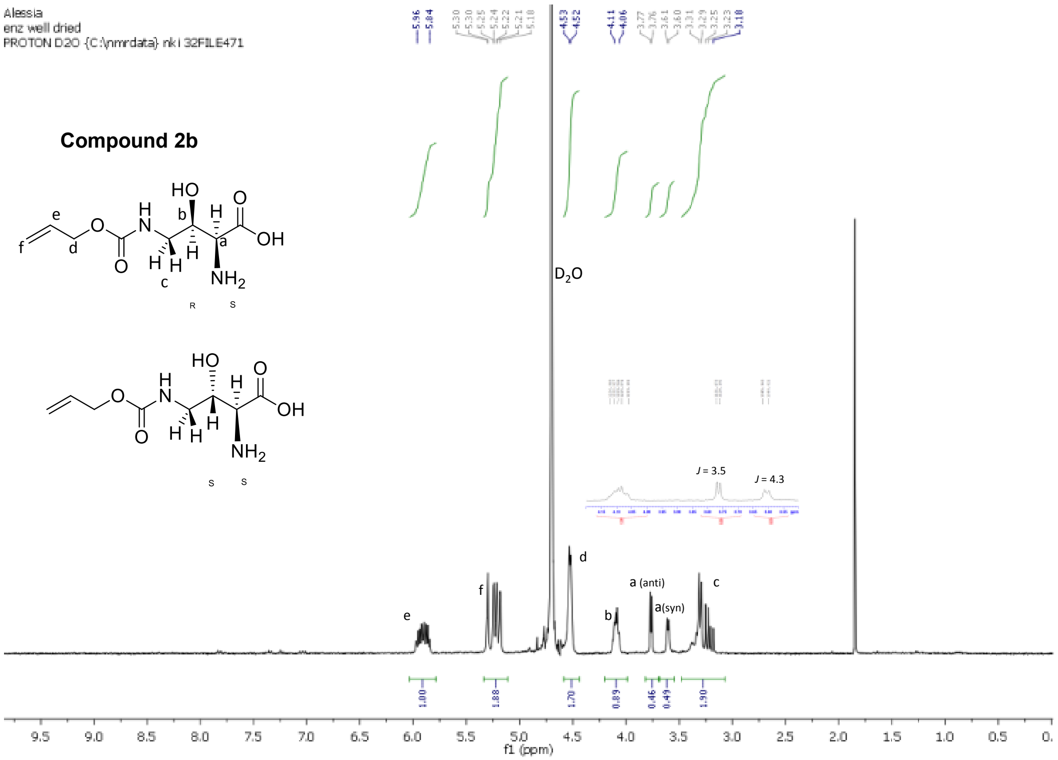

Compound 2b

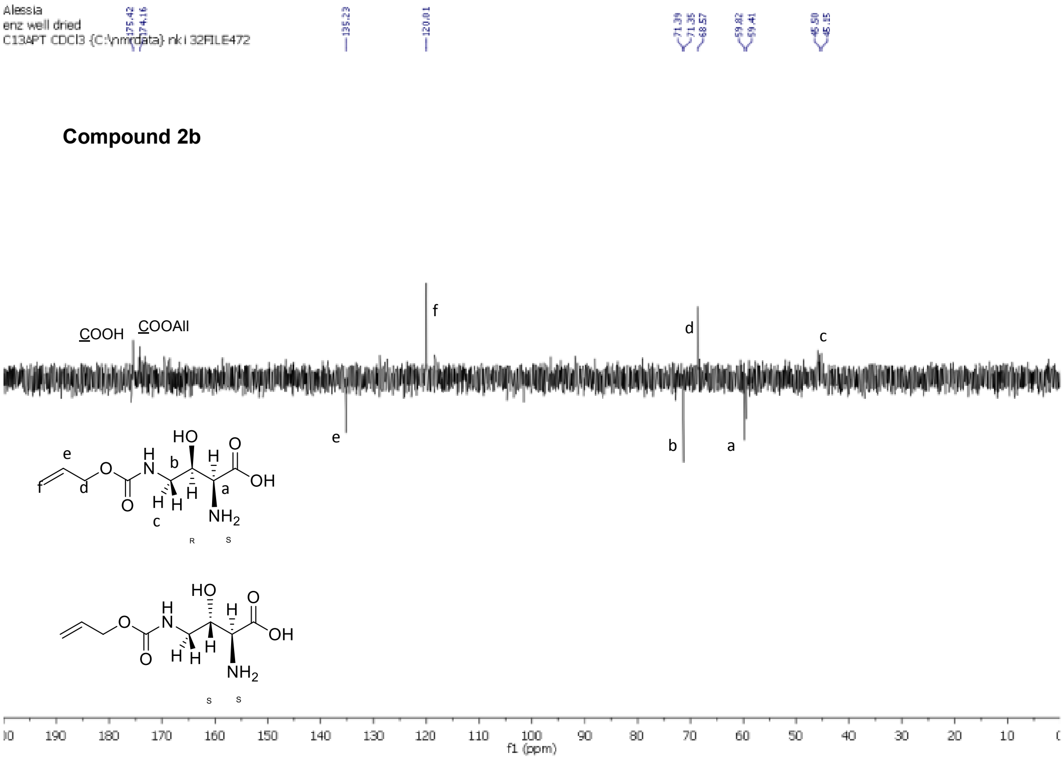

Compound 2b

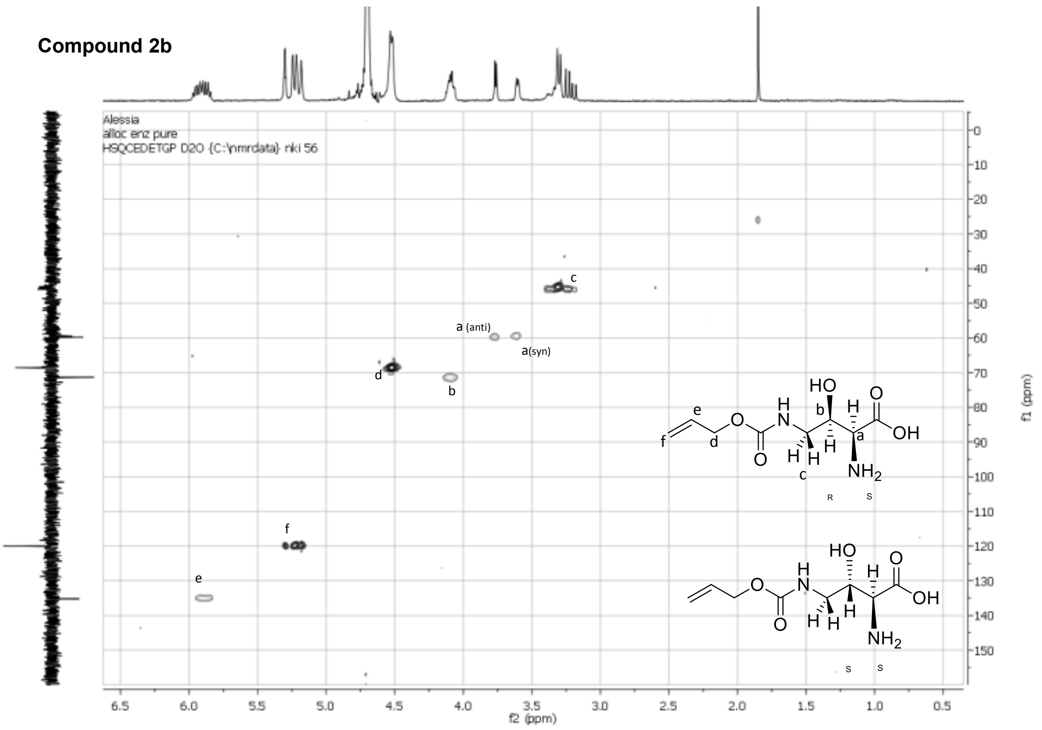
Alessia
alloc enz pure
HSQCEDETGP D2O {C:\nmrdata} nki 56
a (anti)
a(syn)
c
d
b
f
e
f2 (ppm)
f1 (ppm)

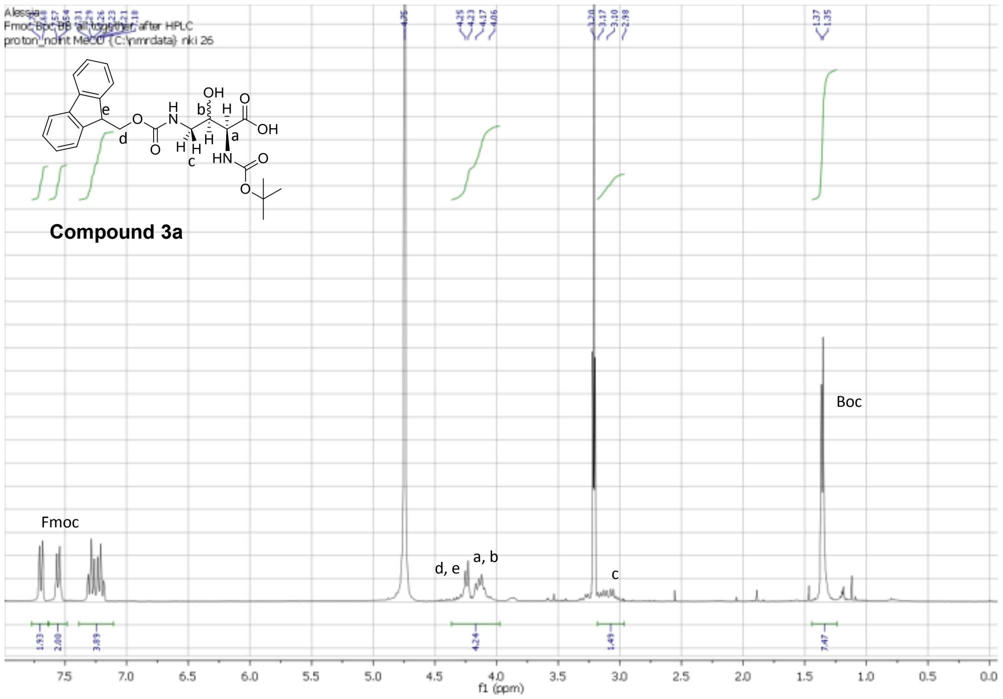
Alessia
Fmoc Boc BB all together after HPLC
proton_nd int MeOD {C:\nmrdata} nki 26
Compound 3a
Fmoc
d, e
a, b
c
Boc
f1 (ppm)

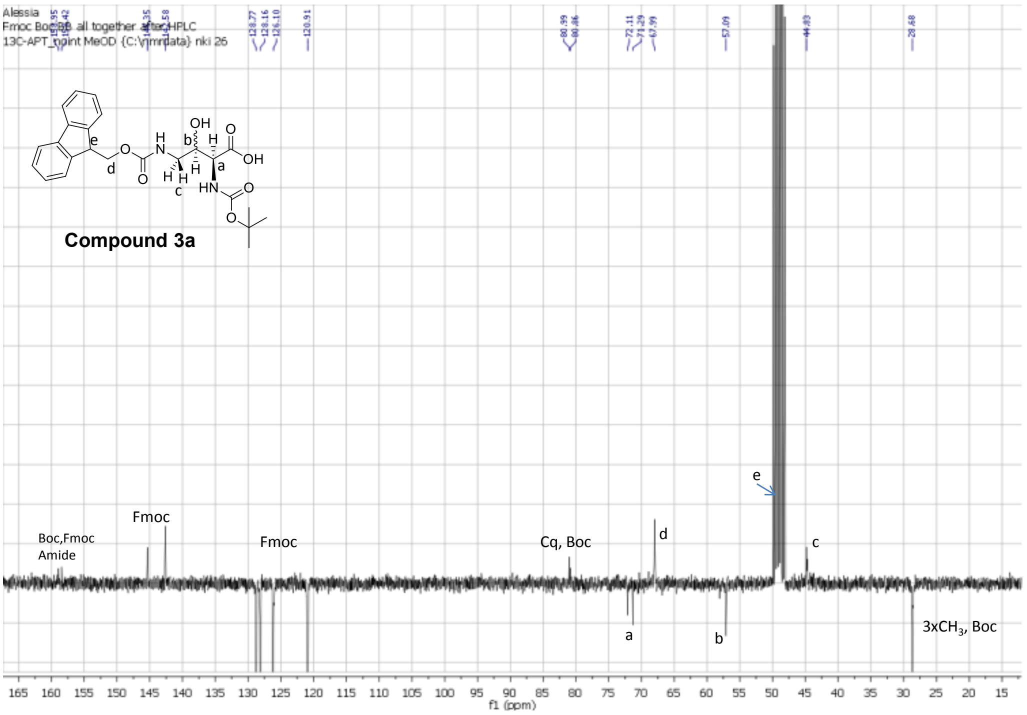
Alessia
Fmoc Boc BB all together after HPLC
13C-APT_point MeOD {C:\nmrdata} nki 26
Compound 3a
Boc,Fmoc
Amide
Fmoc
Fmoc
Cq, Boc
d
e
c
a
b
3xCH3, Boc
f1 (ppm)

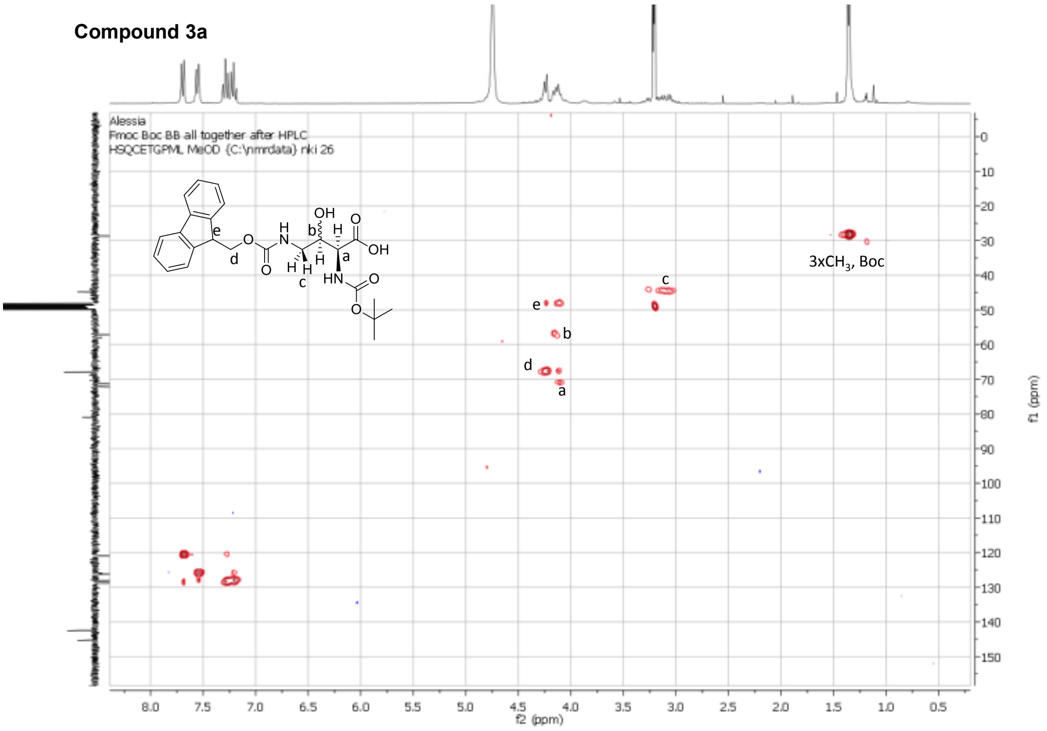
Compound 3a
Alessia
Fmoc Boc BB all together after HPLC
HSQCETGPML MeOD {C:\nmrdata} nki 26
3xCH3, Boc
c
e
b
d
a
f2 (ppm)
f1 (ppm)

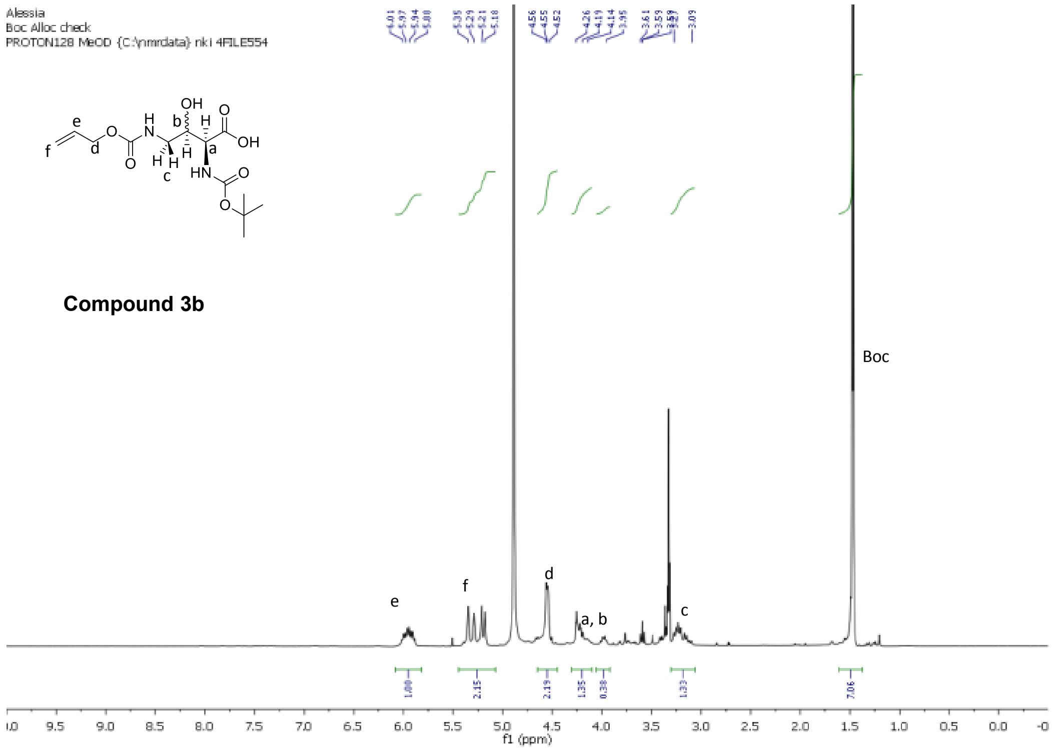
Alessia
Boc Alloc check
PROTON128 MeOD {C:\nmrdata} nkl 4FILE554
Compound 3b
Boc
e
f
d
a, b
c
f1 (ppm)

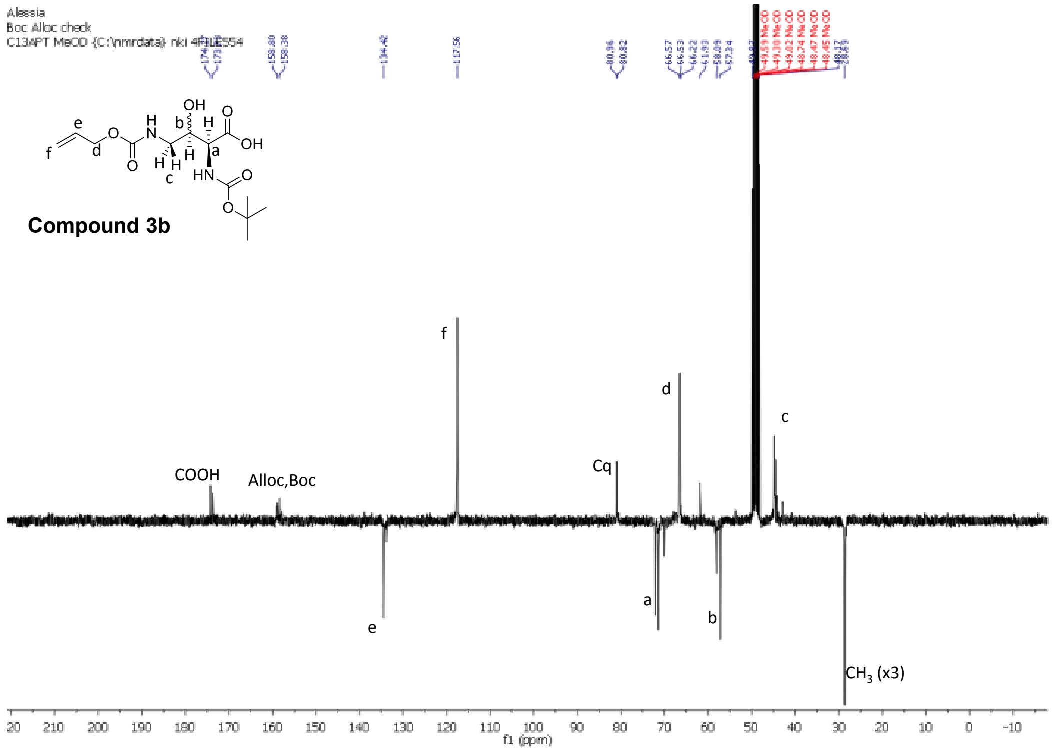
Alessia
Boc Alloc check
C13APT MeOD {C:\nmrdata} nkl 4FILE554
Compound 3b
f
d
c
Cq
COOH
Alloc,Boc
e
a
b
CH3 (x3)
f1 (ppm)

Compound 3b

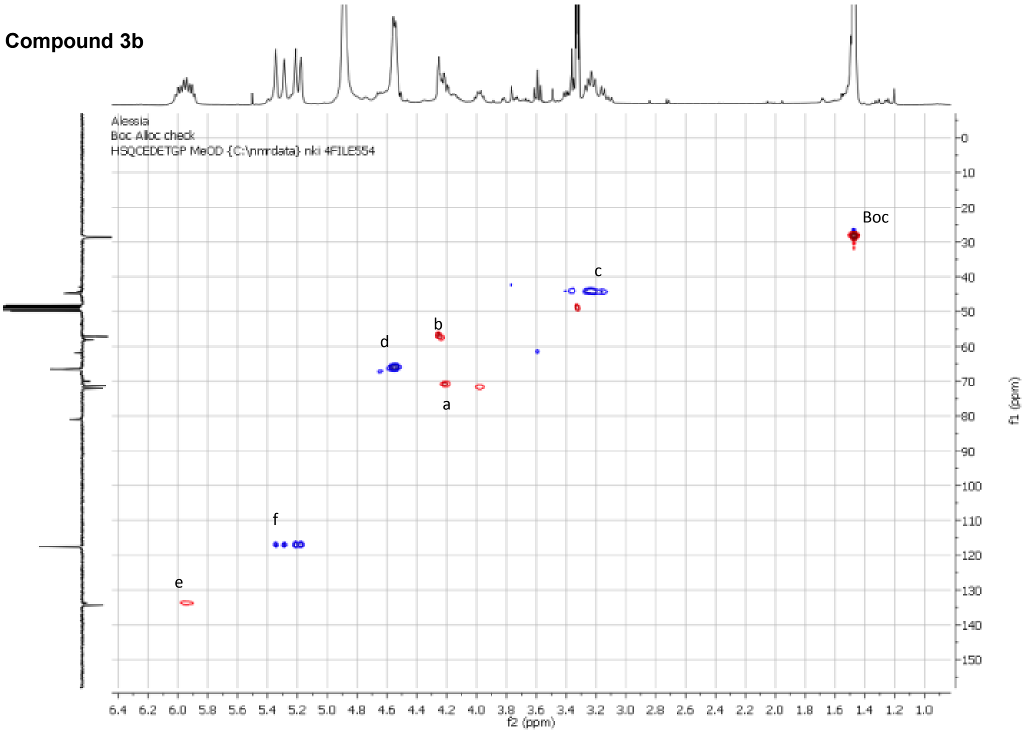

Compound 6

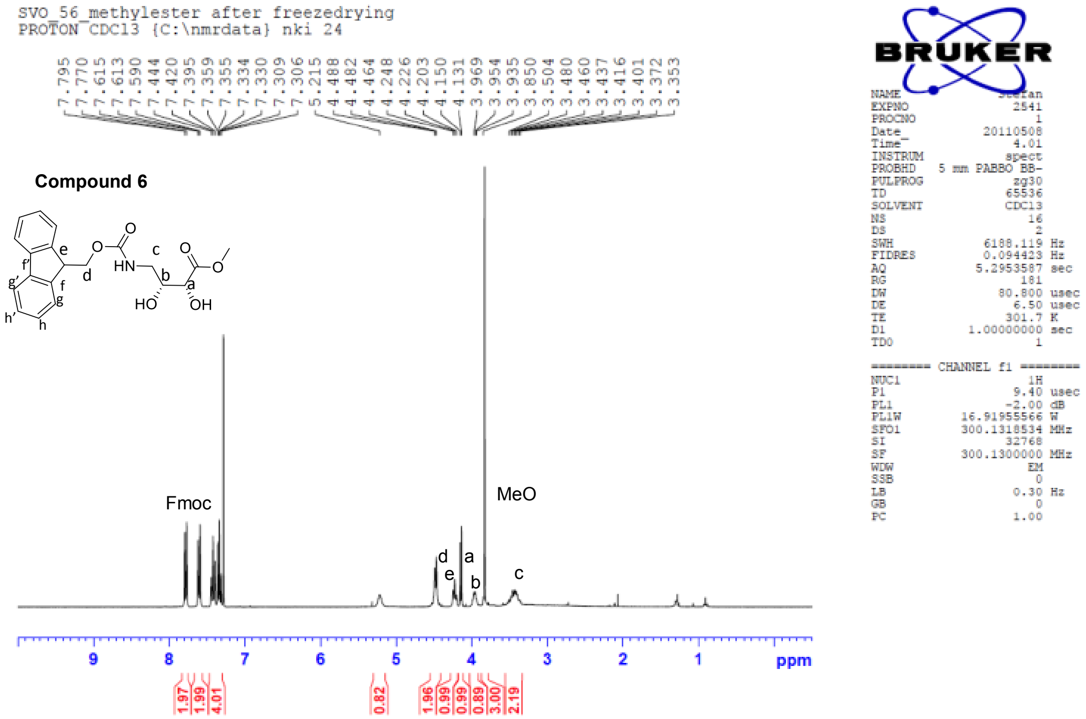

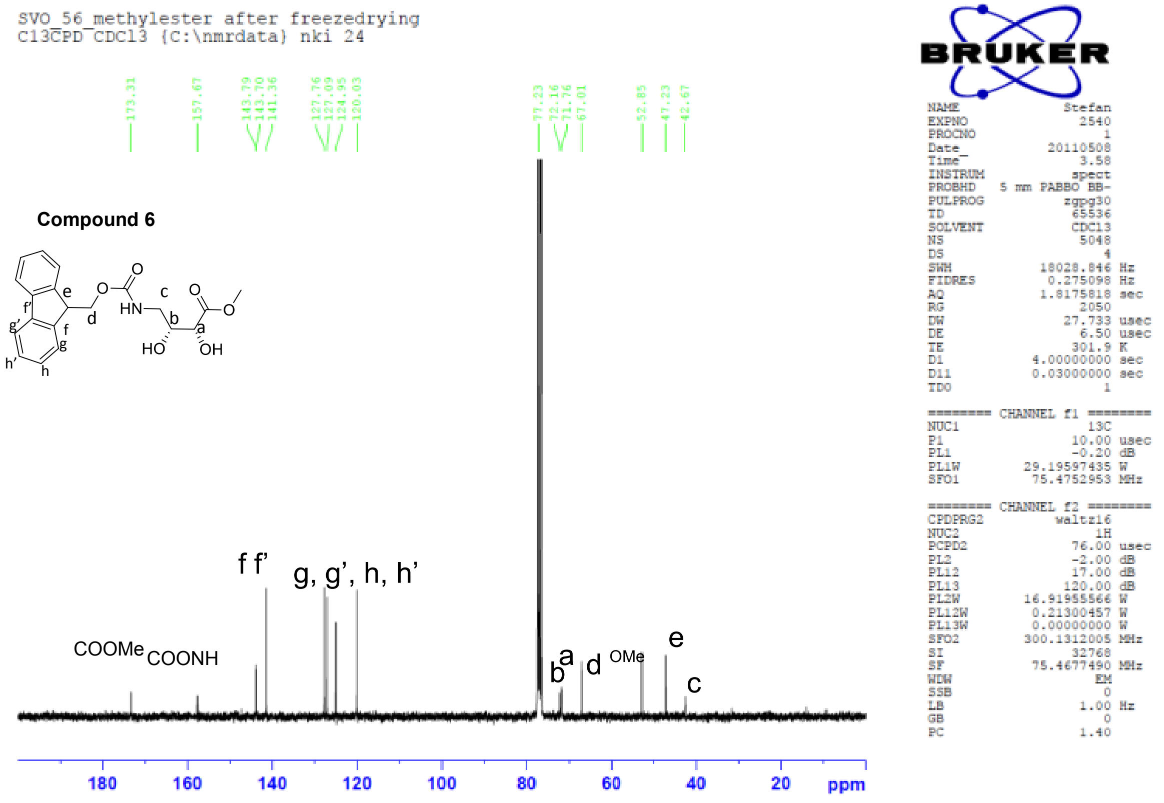
SVO_56_methylester after freezedrying
C13CPD CDCl3 {C:\nmrdata} nki 24
173.31
157.67
143.79
143.70
141.36
127.76
127.09
124.95
120.03
77.23
72.16
71.76
67.01
52.85
47.23
42.67
Compound 6
f f'
g, g', h, h'
COOMe
COONH
a
b
d
OMe
e
c
180
160
140
120
100
80
60
40
20
ppm
BRUKER
NAME Stefan
EXPNO 2540
PROCNO 1
Date_ 20110508
Time 3.58
INSTRUM spect
PROBHD 5 mm PABBO BB-
PULPROG zgpg30
TD 65536
SOLVENT CDCl3
NS 5048
DS 4
SWH 18028.846 Hz
FIDRES 0.275098 Hz
AQ 1.8175818 sec
RG 2050
DW 27.733 usec
DE 6.50 usec
TE 301.9 K
D1 4.00000000 sec
D11 0.03000000 sec
TD0 1
======== CHANNEL f1 ========
NUC1 13C
P1 10.00 usec
PL1 -0.20 dB
PL1W 29.19597435 W
SFO1 75.4752953 MHz
======== CHANNEL f2 ========
CPDPRG2 waltz16
NUC2 1H
PCPD2 76.00 usec
PL2 -2.00 dB
PL12 17.00 dB
PL13 120.00 dB
PL2W 16.91955566 W
PL12W 0.21300457 W
PL13W 0.00000000 W
SFO2 300.1312005 MHz
SI 32768
SF 75.4677490 MHz
WDW EM
SSB 0
LB 1.00 Hz
GB 0
PC 1.40

Compound 6

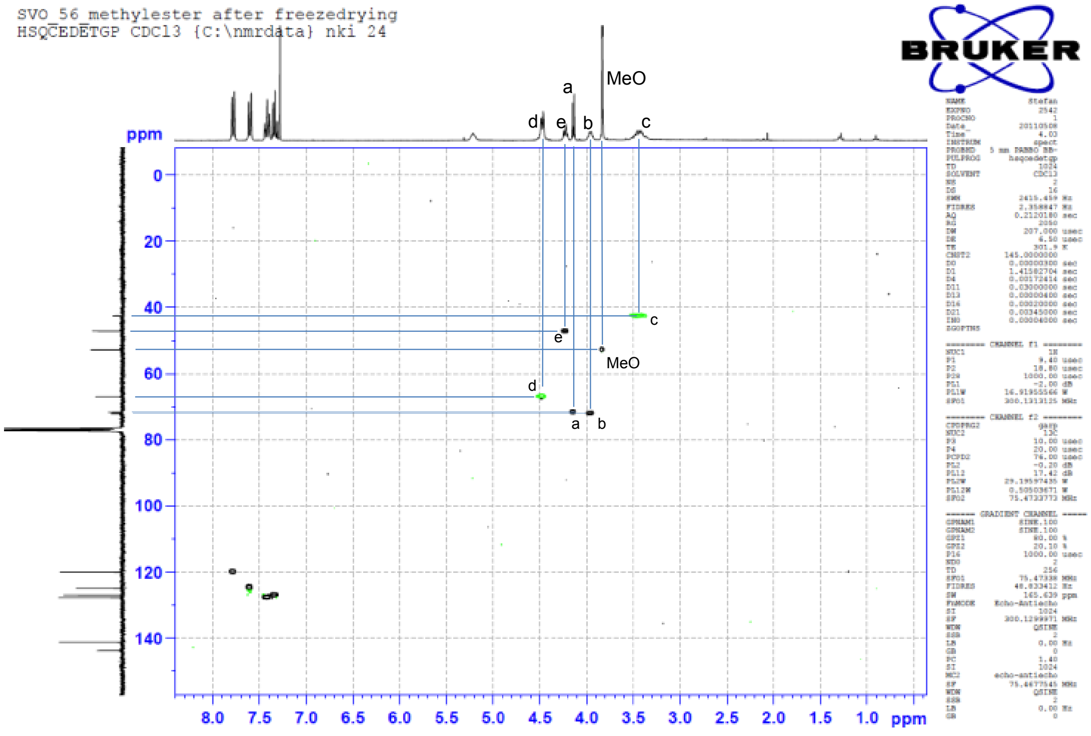
SVO_56_methylester after freezedrying
HSQCEDETGP CDCl3 {C:\nmrdata} nki 24
BRUKER
MeO
a
d
e
b
c
ppm
0
20
40
60
80
100
120
140
8.0
7.5
7.0
6.5
6.0
5.5
5.0
4.5
4.0
3.5
3.0
2.5
2.0
1.5
1.0
ppm

Compound 7

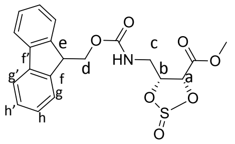

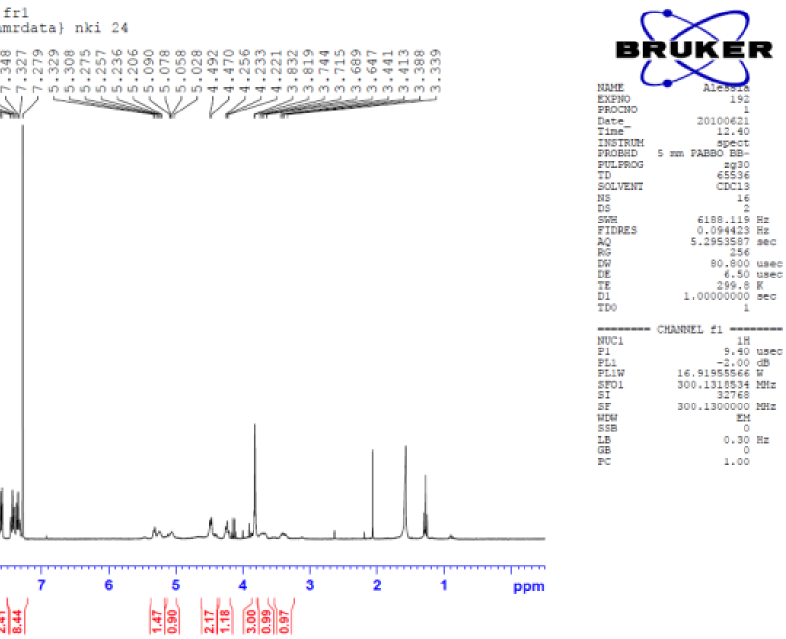

Compound 8

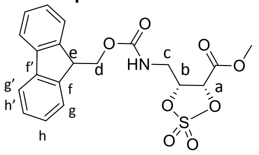

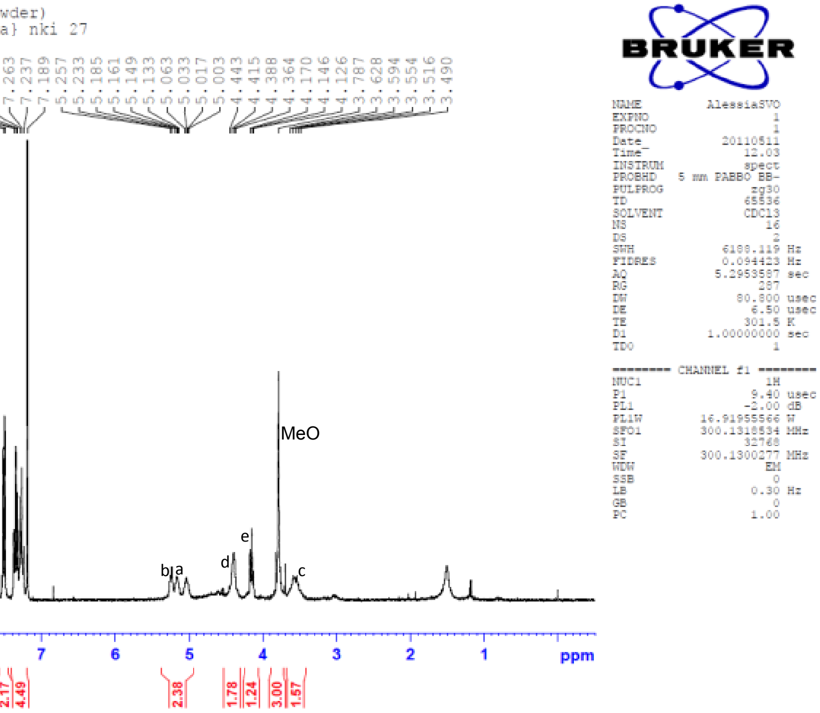

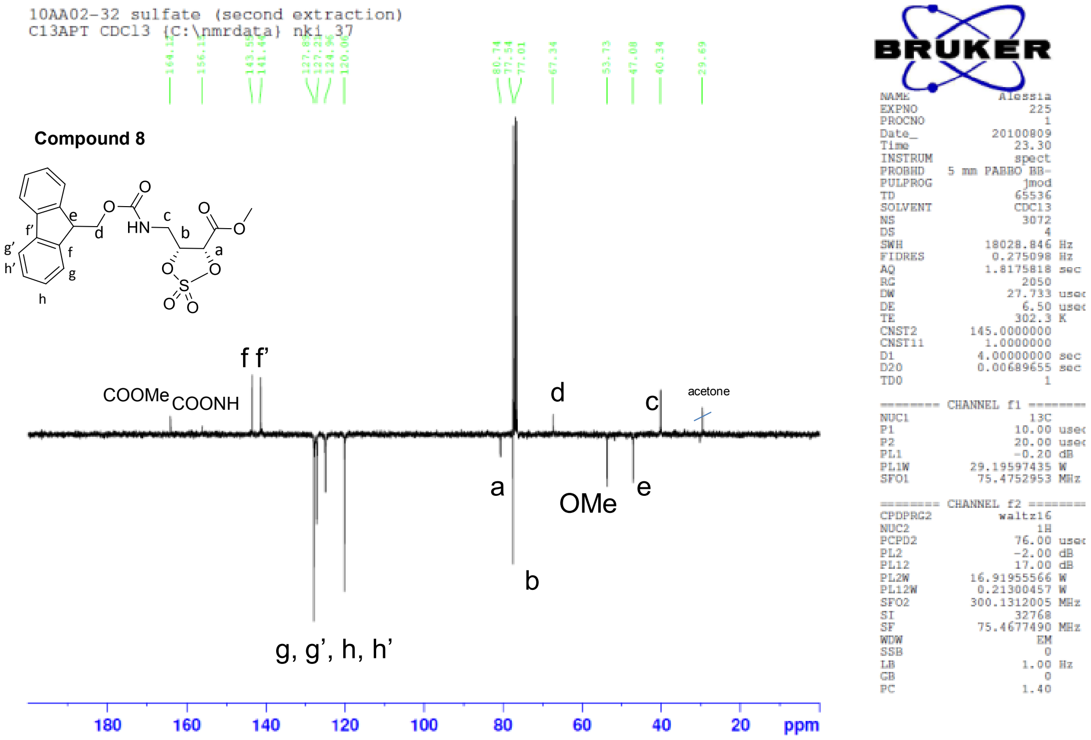

10AA02-32 sulfate (second extraction)
C13APT CDCl3 {C:\nmrdata} nki 37
Compound 8
COOMe
COONH
f f'
d
c
acetone
a
OMe
e
b
g, g', h, h'
ppm
BRUKER

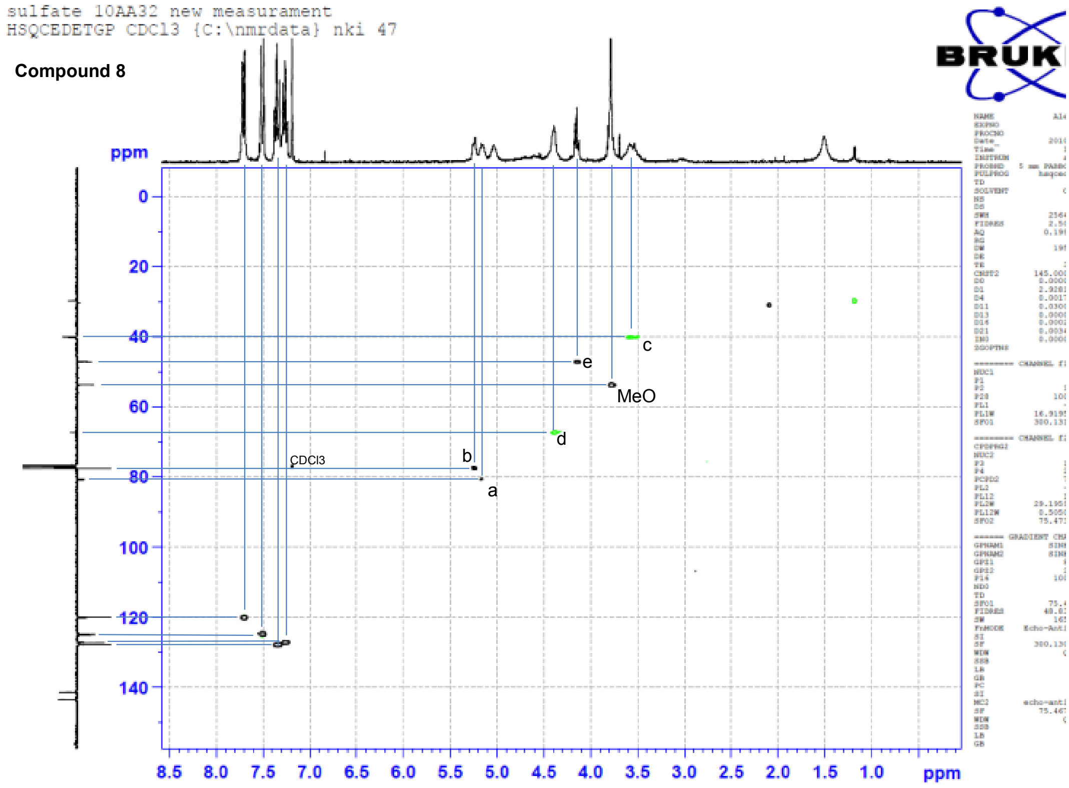

sulfate 10AA32 new measurement
HSQCEDETGP CDCl3 {C:\nmrdata} nki 47
Compound 8
ppm
c
e
MeO
d
b
CDCl3
a
ppm

Compound 9

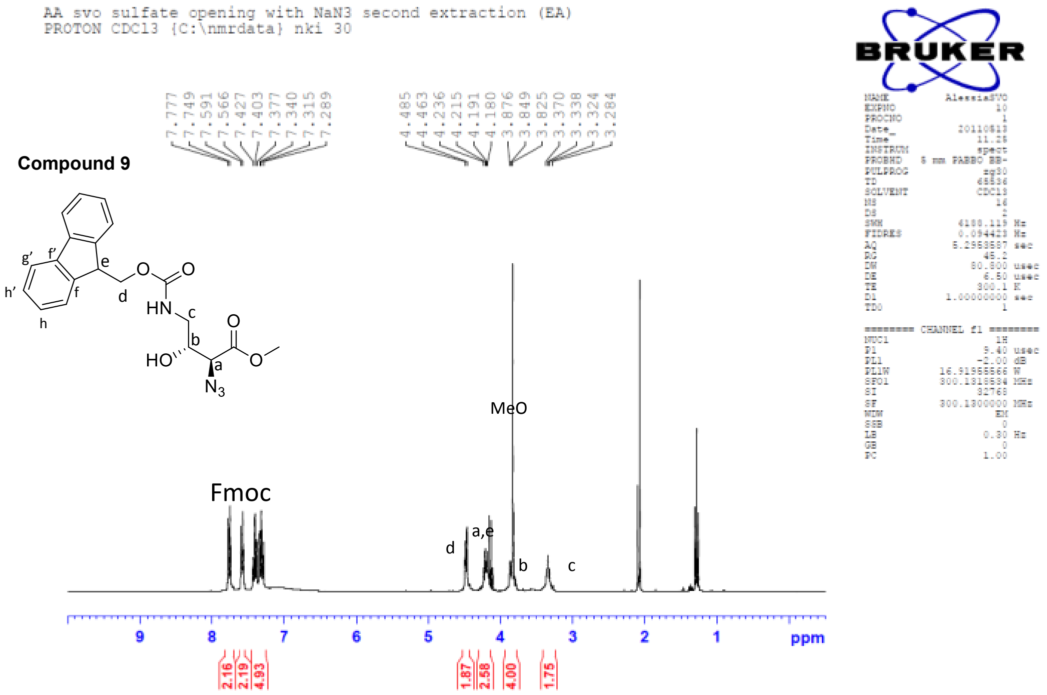

Compound 9

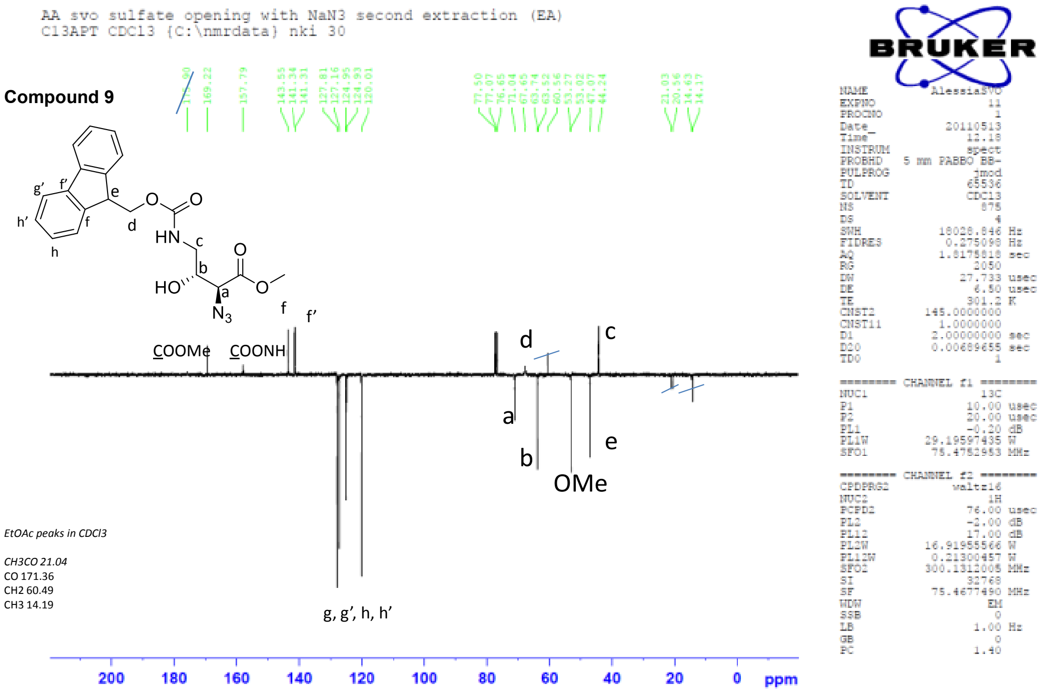

Compound 9

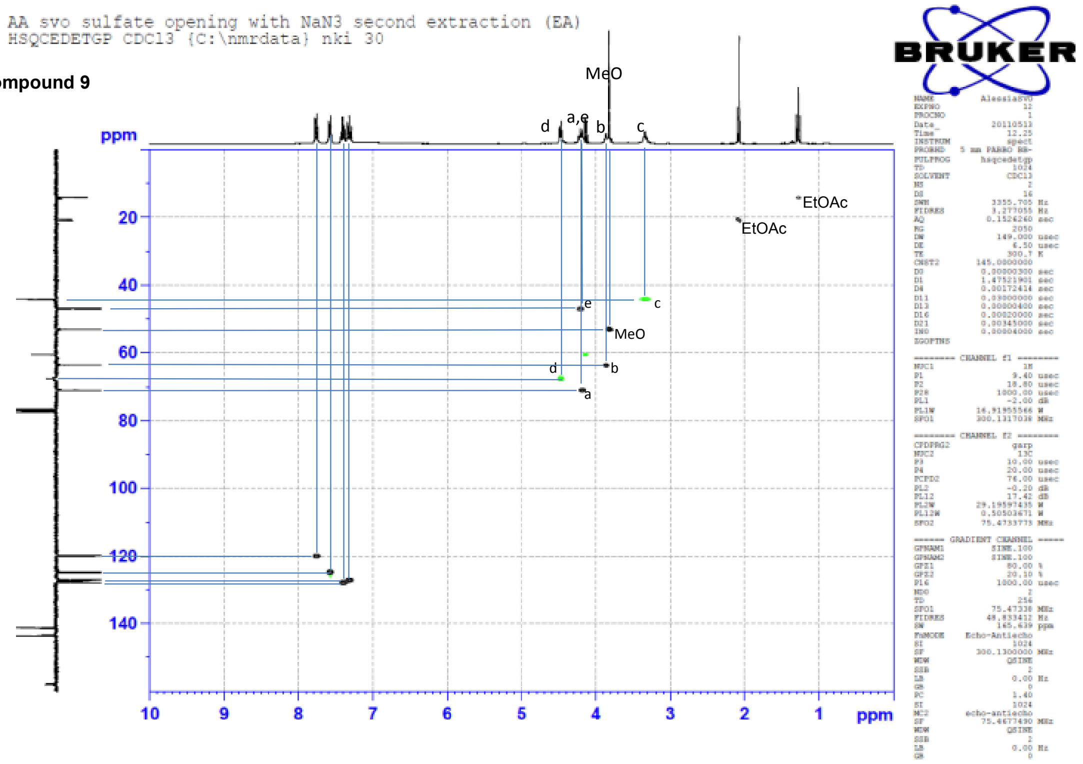

Compound 10

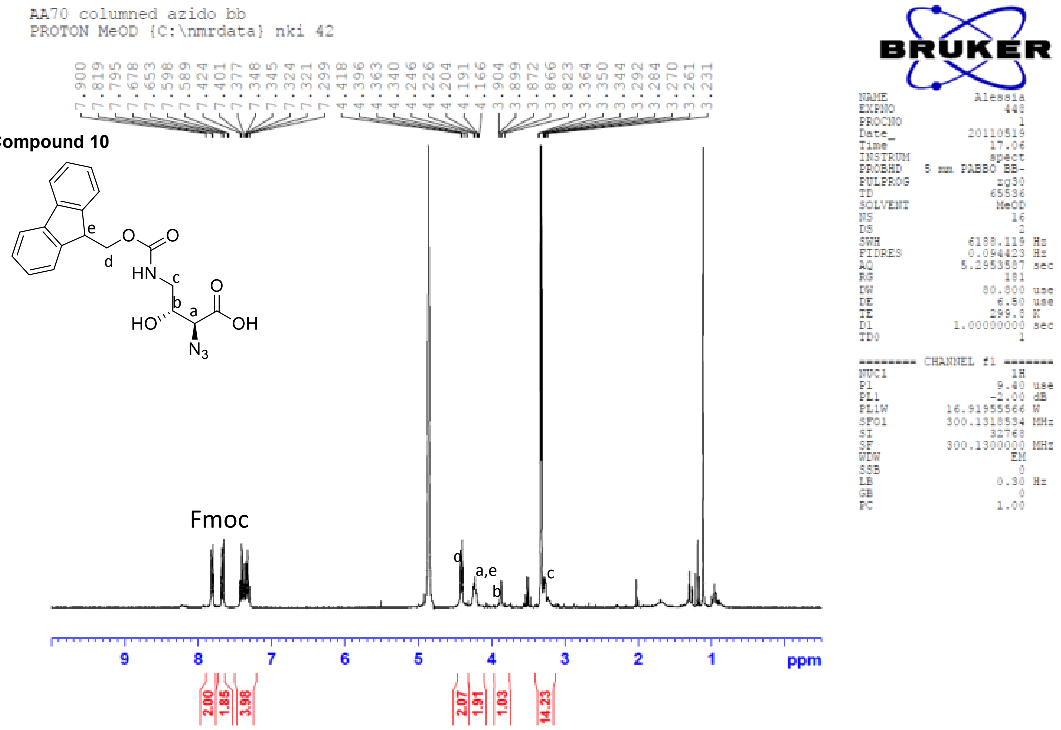

Compound 10

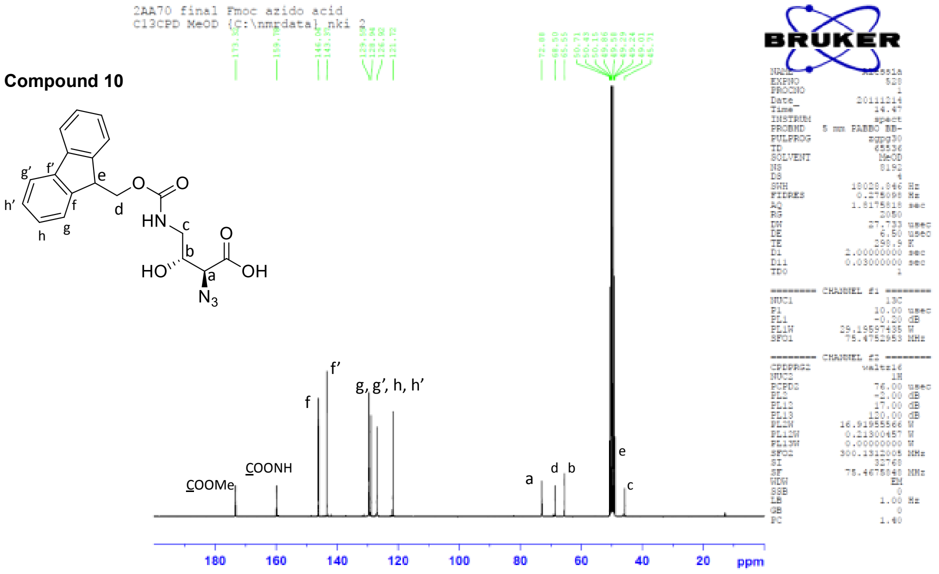

Compound 10

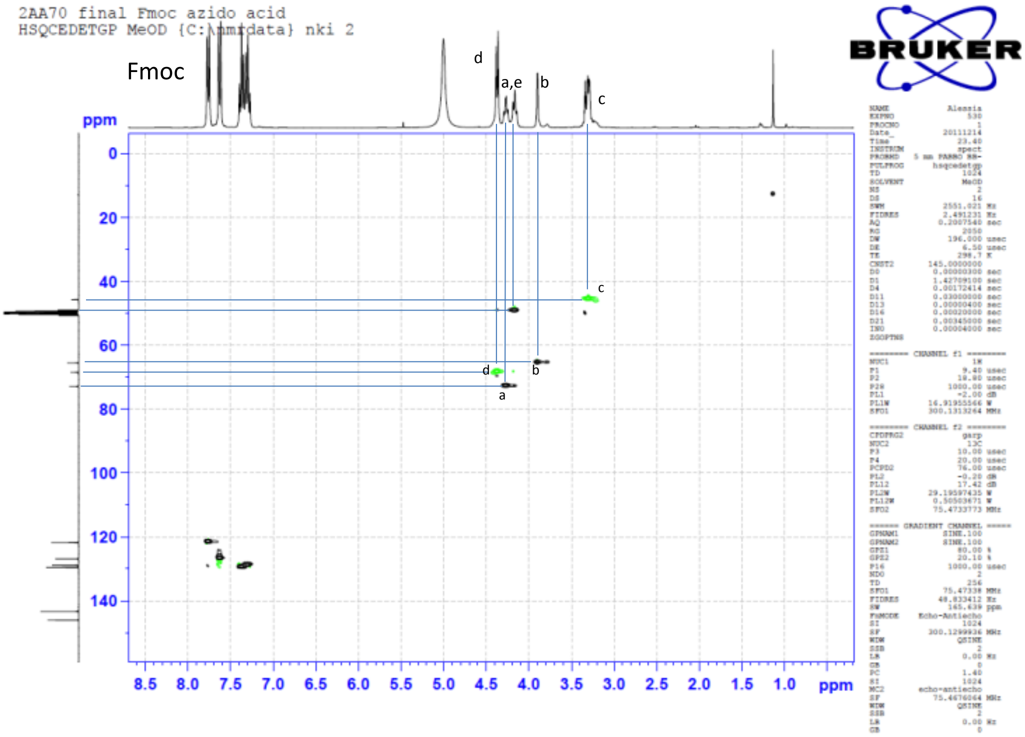

Supplement: Supplementary file 1 [file cbic0014-0123-SD1.pdf]
